# Long-term conservation agriculture with optimum nitrogen fertilization improves soil phosphorus availability

**DOI:** 10.1371/journal.pone.0333013

**Published:** 2025-09-24

**Authors:** Nusrat Jahan Mumu, Sunjana Akter, Afsana Mimi Eiti Mony, Jannatul Ferdous, Nushaiba Atiq Taima, Most. Khatiza Khatun, Md. Mofizur Rahman Jahangir

**Affiliations:** 1 Department of Soil Science, Khulna Agricultural University, Khulna, Bangladesh; 2 Department of Soil Science, Bangladesh Agricultural University, Mymensingh, Bangladesh; ICAR National Bureau of Soil Survey & Land Use Planning, INDIA

## Abstract

Understanding the interactive effects of conservation agriculture (CA) and nitrogen (N) fertilization on soil phosphorus (P) dynamics is critical for sustainable nutrient management. However, information on the dynamics and availability of P pools under the combination of long-term CA practices with N fertilization is limited. This study aimed to evaluate the long-term impacts of CA and different N rates on labile, moderately labile, and non-labile P fractions in a wheat (*Triticum aestivum*)-mungbean (*Vigna radiata*)-rice (*Oryza sativa* L) rotation after the 36th consecutive crop. The field experiment was conducted in a split-split plot design with two tillage systems (conventional tillage, CT; strip tillage, ST), two residue levels (lower residue, LR (15 cm); higher residue, HR (30 cm)), and six nitrogen (N) rates (N0, N60, N80, N100, N120, N140 representing 0%, 60%, 80%,100%, 120% and 140%, respectively of the recommended dose). The ST-HR significantly enhanced labile-P availability. Soluble-P, and NaHCO_3_-Po (organic) increased by 4% and 35% under ST compared to CT, and by 259% and 26% under HR compared to LR, respectively. While HR enhanced most soil P fractions, higher N rates (>N100) tended to decrease labile-Pi (inorganic) fractions by up to 45% suggesting a potential decline in plant-available P. The NaHCO_3_-Pi was higher in HR coupled with lower N rate (≤N100). Optimum N rate under CA aided in raising the NaHCO_3_-Po fractions. Moderately labile-P fractions were higher in ST-HR with increased N rate (>N100). Acid-P increased by 38% under ST, with complex interactions observed across treatments. Acid-P was consistently higher in ST-HR coupled with lower N rate (≤N100) while residual-P was higher in ST-LR coupled with same N rate. The interactions among tillage, residue, and N were significant for most P pools highlighting the synergistic effects of CA and N management. Overall, ST-HR-N100 was found to optimize P availability and minimize non-labile P buildup, offering a balanced P dynamics for improved soil fertility and sustainable crop production.

## Introduction

Soil phosphorus (P) availability is highly sensitive to long-term agronomic practices. Conventional intensive cropping practices, including continuous tillage, monocropping, reliance on chemical fertilizers, and inadequate residue management often accelerate P fixation and deplete labile P pools, leading to declining soil fertility [1,2]. In contrast, conservation agriculture (CA) practices which include minimum soil disturbance, crop residue addition, and rotation with diversified crops can contribute to enhanced P cycling and availability [3,4]. Crop residues play a dual role; they contribute to organic P (Po) pools through organic matter inputs and influence P mineralization depending on their C:P ratios and decomposition dynamics [5,6]. In particular, conservation tillage and crop residue retention have shown potential to increase the availability of labile P fractions by reducing soil erosion and P fixation [4–6].

In agricultural soils, P exists in two broad forms: organic P (Po) and inorganic P (Pi), each of which can be further divided into fractions based on their availability-labile (solution P, NaHCO_3_-Pi, and NaHCO_3_-Po), moderately-labile (NaOH-Pi, NaOH-Po), and non-labile (acid P and residue P) P fractions [7–9]. The equilibrium between these fractions, especially between labile and moderately labile P, governs the short-term dynamics of P supply to crops [10,11]. Plant-available P is largely confined to the labile pool, which is typically small due to the strong tendency of P to bind with soil particles, resulting in fixation [8,11], and over 90% of total soil P is typically found in insoluble and fixed forms [12], limiting its availability and thus crop productivity. Moreover, nitrogen (N) fertilization can mediate the P cycle by affecting microbial-mediated processes, soil pH, and organic matter turnover [2]. Excessive application of ammonium-based fertilizers like urea can acidify the soil, thereby increasing P sorption and reducing labile Pi availability [13]. However, when optimum N is applied under CA conditions, it can stimulate microbial activity and enhance the mineralization of Po, thus increasing Pi availability [2,14]. This N–P interaction is highly context-specific, depending on soil type, cropping system, and climatic conditions. Therefore, location and crop-specific assessments of P behavior in response to N fertilization is crucial for balanced nutrient management and crop production.

Previous research has identified that soils in the Old Brahmaputra Floodplain (AEZ-9) contain higher levels of Po than Pi, making organic P management critical for sustained productivity [15]. Long-term cereal cultivation under varying tillage, residue incorporation and fertilization regimes may lead to significant changes in distribution and availability of different soil P fractions over time in this region. Conservation agriculture practices especially surface residue retention and reduced tillage promote the stratification of P in the upper soil layers, where residues decomposition and root density are highest [16–19]. This stratification has important implications on nutrient uptake, particularly in rainfed or shallow-rooted cropping systems where surface Pi is more accessible, but may limit deeper root access under drought or moisture-stress conditions [20]. Soil P stratification has some other environmental and agronomic implications such as loss of P through runoff and leaching and eutrophication of lake [21,22]. Thus, understanding the extent and functional implications of P stratification is essential for optimizing P use efficiency and crop productivity under CA. Despite its importance, there remains a lack of data and the extent of P availability in crop root zone under CA and how it interacts with N fertilization to influence the distribution and transformation of P fractions over time. Addressing these knowledge gaps is essential for designing nutrient management strategies that utilize CA, optimize P availability, minimize fixation, and sustain productivity under long-term cereal-based cropping system in tropical areas.

Most previous studies have focused either on total P content or crop response, without detailing the shifts among P fractions that determine the availability. Older fractionation methods such as those developed by Chang and Jackson [23], Hingston et al. [24] have been largely replaced by modified sequential extraction techniques [25,26], which allow for a more precise understanding of labile, moderately labile, and non-labile P pools [27]. Maniruzzaman et al. [28] used modified Hedley procedure [25] to determine soil P fractionations in calcareous soils and found that short-term CA practices, particularly increased crop residue retention, significantly elevated almost all P fractions and enhance overall P stocks in topsoil. Reduced tillage was found having smaller yet notable effects by increasing inorganic P fractions in upper layer of soils highlighting residue retention as the dominant driver of P fraction shifts [28]. Our research intends to extend these findings through long-term CA practices and N fertilizer application in a field experiment, specifically within South Asian cereal-based systems, aiming to clarify how these practices influence the turnover and availability of distinct P pools over time.

Moreover, the synergistic effects of CA components such as minimal soil disturbance and residue retention can improve soil physical properties, increase organic matter inputs, and promote microbial activity [29–31]. These factors, in turn, stimulate the mineralization of Po and reduce P adsorption by stabilizing soil aggregates and modifying the chemical environment of the rhizosphere [32–34]. Retaining crop residues in combination with reduced tillage can lessen soil P fixation, increase labile P content, and promote the development of Po and phosphatase-mediated mineralization [35]. The conversion of labile P forms into moderately available and non-labile forms can be facilitated by N fertilization through various soil mechanisms [14]). Balanced N fertilization can enhance microbial biomass and enzymatic activity, thereby facilitating the conversion of Po to plant-available Pi forms. However, excessive or imbalanced N inputs can acidify the soil and increase P sorption, converts labile Pi into recalcitrant P or moderately accessible P [36,37]. The present study hypothesizes that CA combined with optimal N fertilization will enhance soil P availability for plant uptake by increasing labile and organic P fractions while minimizing P fixation. In addition, the positive interaction of CA with N inputs can be leveraged for sustainable nutrient management influencing the transformation and mobilization of soil P. The objectives of this study were (i) to assess the long-term effects of CA with varying N fertilization on the dynamics and distribution of soil P fractions in a cereal-based cropping system; and ii) to identify the optimum N fertilization rate that minimizes P fixation and enhances plant-available P fractions under CA practices.

## Materials and methods

### Site description

The research was conducted on the Soil Science Field Laboratory (24°71.60’N, 90°42.51’E) at Bangladesh Agricultural University, Mymensingh in Bangladesh. The field site is located on the Old Brahmaputra Floodplain soil, and is classified as non-calcareous dark grey floodplain soil under the Sonatala series. It has a silty loam texture, is neutral in pH (6.5), and poorly drained. With an average yearly temperature of 26° C, 1800 mm of rainfall, and 85% relative humidity, the area experiences sub-tropical monsoon weather (Weather station, BAU). Farmers in Mymensingh region commonly follow the wheat-fallow-rice cropping pattern but, in the experiment, we included mungbean which is a short duration leguminous crop, as a CA practice. Thus, the experiment followed a wheat (BARI hybrid maize-14) – mungbean (BARI Mung-6) –rice (BRRI-71) cropping pattern which was continued at this location for 12 years.

### Experimental design and treatment applications

In the experimental site, CA (minimum tillage, soil cover residue, and adding short-duration legumes to crop rotation) was implemented as a long-term practice since 2011. There were three sets of treatments in the split-split plot design of the experiment: 1) conventional tillage (CT) and strip tillage (ST- only the rows (2–3 cm) were tilled and the areas (20 cm) in between the rows were left untilled, tilled rows were used for planting crops) were assigned to the main plots; 2) crop residue retention was assigned to the sub-plots (higher residue, HR: 30 cm of rice and wheat residue + 100% mungbean residue, by height; lower residue, LR: 15 cm of rice and wheat residue + 100% mungbean residue); and 3) Nitrogen fertilizer dose: N0: 0% of RD (recommended dose); N60: 60% of RD; N80: 80% of RD; N100: 100% of RD; N120: 120% of RD; N140: 140% of RD were assigned to sub-sub plots with three replications. Recommended fertilizer dose was followed from the national fertilizer recommendation guide for the location-specific test crops [38]. The 100% RD for cultivating wheat, mung bean and rice at the experimental location was 100 kg N/ha, 20 kg N/ha and 120 kg N/ha, respectively.

Each sub-sub plot was 13 m × 3 m and had a dividing bund around each plot. Urea, triple super phosphate, muriate of potash, gypsum, magnesium sulphate, zinc sulphate and boric acid were used for wheat, mungbean and rice as sources of N, P, K, S, Mg, Zn and B, respectively and the fertilizers were applied following the national fertilizer recommendation dose [38] for this region known as Agro-ecological Zone (AEZ) 9. During the final land preparation, a basal dose of every fertilizer had been put to every plot, with the exception of urea during rice and wheat cultivation. Urea was applied to each plot in three equal split applications in wheat and rice. Moreover, intercultural practices including irrigation, weeding, insect and disease management were done aligned with the cultural practices for Bangladesh AEZ-9.

### Soil sample collection, processing and analysis

**Fig 1:**
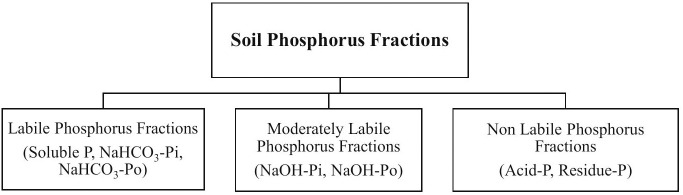
Different soil phosphorus fractions according to lability.

**Fig 2:**
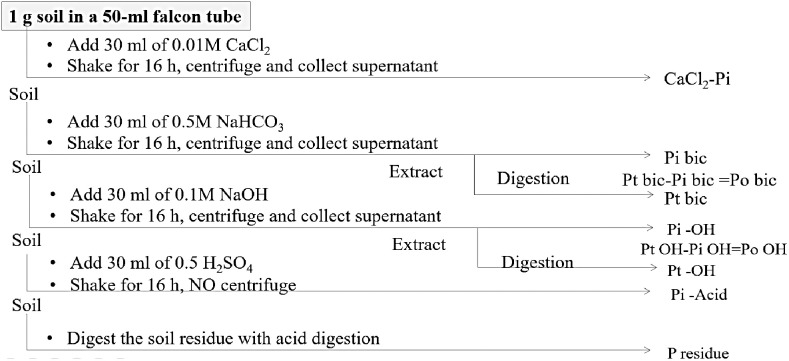
Soil phosphorus fractionations procedure [41].

Composite soil samples were collected from each plot in 2023 after thirty-six consecutive crops (12 years following the repeated cropping sequence and CA). Soil samples were collected after crop harvesting using an auger from five different spots per plot randomly at 0–15 cm soil depth. The collected soil samples were mixed thoroughly to make a composite soil sample, air-dried at room temperature, passed through a 2 mm sieve, and stored until further analysis. One gram of finely grounded soil sample (0.2 mm) was extracted sequentially for soil P fractionations (Fig 1) following the method described by Hedley [26], with a further modification involving the final soil digestion by nitric acid and perchloric acid [39]. The sequential extractions of seven P fractions including solution P, NaHCO_3_-P (Pi and Po), NaOH-P (Pi and Po), acid P and residual P were done (Fig 2).

Solution P was extracted by 0.01 M CaCl_2_ for inorganic P fraction. Soil NaHCO_3_-P fraction was extracted by 0.5 M NaHCO_3_ and NaHCO_3_-Po (organic) were estimated by deducting NaHCO_3_-Pi (inorganic) from the total NaHCO_3_-extractable P following H_2_SO_4_ and H_2_O_2_ digestion. NaOH-P fraction was extracted by 0.1 M NaOH. After being digested with H_2_SO_4_ and H_2_O_2_, NaOH-Pi was deducted from the total NaOH-extractable P to determine NaOH-Po. Acid-P was extracted by 0.5 M H_2_SO_4_ and residual-P was extracted by HNO_3_ and HClO_4_ (5:2 ratio). The concentration of P in the extracts and digests was measured using the ammonium molybdate-ascorbic acid method after neutralization (if necessary) and the absorbance of P was determined using a spectrophotometer at 882 nm wavelength [40].

### Statistical analysis

Tillage, residues, and N rate were the fixed factors in a split-split plot (three-way analysis) analysis of variance (ANOVA). To determine the impact and interactions between and among tillage, residue, and N rate treatments, data was statistically analysed using the Jamovi 1.0.0.0. (R Package) software. The comparison of means was tested by the Duncan’s Multiple Range Test (DMRT) and least significant difference (LSD) at the p < 0.05 level using SPSS (IBM SPSS Statistics, version 20, IBM Corp).

## Results

### Effects of management practices on labile phosphorus fractions

Soluble-P content was significantly influenced by the interaction of tillage, residue retention, and nitrogen (N) application rate (P < 0.001, Table 1). The highest soluble-P (13.4 mg kg^-1^) was observed under CT-HR-N100, which was over 12 times higher than the lowest value (1.0 mg kg^-1^) in CT-LR-N120. Soluble-P decreased with increasing N rates beyond N100, particularly under low residue (LR) treatments in either tillage.

**Table 1:** Labile-P fractions in soil (depth: 0-15 cm) under long term conventional (CT) and strip (ST) tillage systems; high residue (HR) and low residue (LR) retention; and six N application rates.

Treatments			Labile Phosphorus Fractions		
Tillage	Residue	Fertilizer	Soluble-P (mg kg^-1^)	NaHCO_3_-Pi (mg kg^-1^)	NaHCO_3_-Po (mg kg^-1^)
CT	HR	N0	3.3 ± 0.01g	44.9 ± 0.25b	18.2 ± 0.45bd
		N60	4.3 ± 0.01f	43.2 ± 0.02c	13.0 ± 0.45ce
		N80	5.5 ± 0.01e	35.9 ± 0.01d	15.6 ± 0.78be
		N100	13.4 ± 0.00a	35.0 ± 0.02d	15.6 ± 0.78be
		N120	11.2 ± 0.01b	25.4 ± 0.01f	13.0 ± 0.45ce
		N140	8.0 ± 0.05c	21.6 ± 0.05h	18.2 ± 0.45bd
	LR	N0	2.4 ± 0.02h	24.2 ± 0.14g	15.6 ± 0.00be
		N60	3.3 ± 0.02g	21.6 ± 0.05h	15.6 ± 0.78bce
		N80	4.2 ± 0.02f	2.4 ± 0.05p	15.6 ± 0.00be
		N100	1.1 ± 0.00k	2.7 ± 0.03op	20.8 ± 0.90bc
		N120	1.0 ± 0.01k	4.8 ± 0.00n	15.6 ± 0.00be
		N140	1.6 ± 0.03ij	3.9 ± 0.01no	18.2 ± 0.45bd
ST	HR	N0	5.5 ± 0.01e	46.0 ± 0.03a	18.2 ± 0.45bd
		N60	11.2 ± 0.01b	30.3 ± 0.02e	30.0 ± 0.00ab
		N80	11.2 ± 0.01b	14.4 ± 0.02j	20.8 ± 0.90bc
		N100	7.9 ± 0.01 cd	13.2 ± 0.03k	36.0 ± 0.00a
		N120	7.5 ± 0.06d	10.5 ± 0.03l	30.0 ± 0.90ab
		N140	5.7 ± 0.03e	10.0 ± 0.03l	28.0 ± 0.78bc
	LR	N0	3.3 ± 0.01g	17.1 ± 0.13i	18.2 ± 0.45bd
		N60	3.2 ± 0.00g	10.8 ± 0.03l	20.8 ± 0.45bc
		N80	1.4 ± 0.03ik	9.8 ± 0.01l	23.4 ± 0.00b
		N100	1.7 ± 0.01ij	6.7 ± 0.01m	20.8 ± 0.45bc
		N120	1.7 ± 0.01i	4.6 ± 0.02n	10.4 ± 0.45de
		N140	1.2 ± 0.07jk	2.9 ± 0.01op	7.8 ± 0.00e
LSD (p < 0.05)			0.04	0.11	0.08
Level of Significance					
Tillage x Residue			^**^	^***^	^*^
Fertilizer x Tillage			^***^	^***^	^**^
Fertilizer x Residue			^***^	^***^	^***^
Fertilizer x Tillage x Residue			^***^	^***^	^***^

CT = conventional tillage; ST = strip tillage; HR = higher residue: 30 cm of rice and wheat residue + 100% mungbean residue, by height; LR = lower residue: 15 cm of rice and wheat residue + 100% mungbean residue; N0 = 0% of recommended dose; N60 = 60% of recommended dose; N80: 80% of recommended dose; N100: 100% of recommended dose; N120: 120% of recommended dose; N140: 140% of recommended dose.

ns,

*,

**,

***indicate significant difference at the level P > 0.05, P < 0.05, P < 0.01, P < 0.001, respectively. Means separated by same lower-case letter (a, b, and c) under each column were not significantly different at p > 0.05 among different treatment combinations.

The interaction effects of tillage, residue, and N levels were significant for the NaHCO_3_-inorganic P (NaHCO_3_-Pi) fraction (P < 0.001, Table 1), ranging from 2.4 to 46.0 mg kg^-1^. The highest value (46.0 mg kg^-1^) was recorded under ST-HR-N0, which was about 90% higher than ST-HR-N100 (13.2 mg kg^-1^). Notably, NaHCO_3_-Pi decreased sharply with increasing N rates under both ST-HR and ST-LR (Table 1). Combination of CT-HR with lower N rates considerably raised the soils’ NaHCO_3_-Pi fraction compared to CT-LR with similar N rates. Likewise, the NaHCO_3_-organic P fractions (NaHCO_3_-Po) varied significantly among the treatment combinations (P < 0.001, Table 1). The ST-HR-N100 treatment showed the highest NaHCO_3_-Po (36.0 mg kg^-1^), about 5 times increase over the minimum value observed under ST-LR-N140 (7.8 mg kg^-1^) treatment. Higher N rates (> N3) tended to decrease NaHCO_3_-Po levels across tillage and residue treatments.

### Effects of management practices on moderately labile phosphorus fractions

Tillage, residue, and N rates significantly influenced NaOH-extractable inorganic P (NaOH-Pi) fractions (P < 0.001, Table 2). The NaOH-Pi ranged from 5.6 to 38.2 mg kg^-1^, with the highest value recorded in the ST-HR-N140 treatment, nearly 7-fold higher than the minimum in CT-HR-N0 treatment combination. The NaOH-Pi fraction was significantly increased with elevated N rates combining with CT-HR, ST-HR and ST-LR treatments. Notably, NaOH-Pi levels in ST consistently outperformed CT under similar residue and N treatments.

**Table 2:** Moderately labile-P fractions in soil (depth: 0-15 cm) under long term conventional (CT) and strip (ST) tillage systems; high residue (HR) and low residue (LR) retention; and six N application rates.

Treatments			Moderately Labile Phosphorus Fractions	
Tillage	Residue	Fertilizer	NaOH-Pi (mg kg^-1^)	NaOH-Po (mg kg^-1^)
CT	HR	N0	5.6 ± 0.01r	20.8 ± 0.45 cg
		N60	8.6 ± 0.02p	18.2 ± 0.45dh
		N80	9.7 ± 0.01o	20.8 ± 0.90 cg
		N100	10.4 ± 0.03o	28.6 ± 0.90bd
		N120	13.4 ± 0.00m	33.8 ± 0.90b
		N140	16.6 ± 0.03k	33.8 ± 0.90b
	LR	N0	7.2 ± 0.05q	15.6 ± 0.78eh
		N60	5.7 ± 0.01r	11.7 ± 0.39fh
		N80	7.7 ± 0.10pq	7.8 ± 0.00h
		N100	17.8 ± 0.05j	10.4 ± 0.45gh
		N120	18.2 ± 0.00j	10.4 ± 0.45gh
		N140	15.2 ± 0.02l	10.4 ± 0.45gh
ST	HR	N0	22.3 ± 0.03g	15.6 ± 0.78eh
		N60	26.9 ± 0.19e	18.2 ± 0.45dh
		N80	33.2 ± 0.09d	31.2 ± 0.78bc
		N100	35.4 ± 0.04c	52.0 ± 0.45a
		N120	36.6 ± 0.03b	54.6 ± 0.78a
		N140	38.2 ± 0.04a	54.6 ± 0.78a
	LR	N0	6.1 ± 0.05r	18.2 ± 0.45dh
		N60	12.4 ± 0.01n	17.1 ± 0.25dh
		N80	13.8 ± 0.01m	20.8 ± 0.90 cg
		N100	19.1 ± 0.04i	23.4 ± 1.35bf
		N120	21.1 ± 0.01h	23.4 ± 1.35bf
		N140	24.2 ± 0.03f	26.0 ± 1.19be
LSD (p < 0.05)			0.09	1.28
Level of Significance				
Tillage x Residue			^***^	ns
Fertilizer x Tillage			^***^	^**^
Fertilizer x Residue			^***^	^***^
Fertilizer x Tillage x Residue			^***^	ns

CT = conventional tillage; ST = strip tillage; HR = higher residue: 30 cm of rice and wheat residue + 100% mungbean residue, by height; LR = lower residue: 15 cm of rice and wheat residue + 100% mungbean residue; N0 = 0% of recommended dose; N60 = 60% of recommended dose; N80: 80% of recommended dose; N100: 100% of recommended dose; N120: 120% of recommended dose; N140: 140% of recommended dose.

ns,

*,

**,

***indicate significant difference at the level P > 0.05, P < 0.05, P < 0.01, P < 0.001, respectively. Means separated by same lower-case letter (a, b, and c) under each column were not significantly different at p > 0.05 among different treatment combinations.

No significant interaction among tillage, residue and N rates was found for NaOH-Po (p > 0.05, Table 2) fractions. The soil NaOH-Po fractions ranged from 7.4 to 54.6 mg kg^-1^ across all treatments. Mean NaOH-Po concentrations under ST were 60% higher than under CT, and HR showed nearly double NaOH-Po concentrations compared to LR treatments. Nitrogen fertilization significantly enhanced NaOH-Po buildup and the highest NaOH-Po (54.6 mg kg^-1^) was observed under ST-HR-N120 and ST-HR-N140, highlighting the role of higher N rates.

### Effects of management practices on non-labile phosphorus fractions

The interaction between tillage, residue level, and N fertilizers significantly influenced the acid-P fractions in soil (P < 0.001, Table 3). The maximum value was observed in ST-HR-N60 (24.2 mg kg^-1^), while the lowest occurred in ST-LR-N120 (2.0 mgkg^-1^), indicating that moderate N levels under HR and ST enhance acid-P accumulation.

**Table 3:** Non labile-P fractions in soil (depth: 0-15 cm) under long term conventional (CT) and strip (ST) tillage systems; high residue (HR) and low residue (LR) retention; and six N application rates.

Treatments			Non-labile Phosphorus Fractions	
Tillage	Residue	Fertilizer	Acid-P (mg kg^-1^)	Residue-P (mg kg^-1^)
CT	HR	N0	7.0 ± 0.09hi	13.3 ± 0.14bf
		N60	10.7 ± 0.12fh	15.8 ± 0.29bd
		N80	10.0 ± 0.00gh	12.5 ± 0.43cf
		N100	14.5 ± 0., e.g.,	15.6 ± 0.000be
		N120	8.1 ± 0.08hi	19.2 ± 0.38b
		N140	10.0 ± 0.00gh	9.7 ± 0., e.g.,
	LR	N0	7.5 ± 0.00hi	15.8 ± 0.29bd
		N60	14.5 ± 0., e.g.,	15.8 ± 0.29bd
		N80	10.5 ± 0.15fh	15.9 ± 0.30bd
		N100	16.2 ± 0.03be	11.6 ± 0.06 dg
		N120	15.0 ± 0.15df	17.5 ± 0.01bc
		N140	24.0 ± 1.31a	12.4 ± 0.03cf
ST	HR	N0	21.0 ± 0.26ab	19.2 ± 0.14b
		N60	24.2 ± 0.14a	14.2 ± 1.15bf
		N80	20.0 ± 0.00ac	9.2 ± 0.29fh
		N100	18.4 ± 0.04be	11.7 ± 0.38 cg
		N120	15.5 ± 0.39ce	15.8 ± 1.04bd
		N140	20.5 ± 0.09ab	5.8 ± 0.14gh
	LR	N0	20.0 ± 0.02ac	34.2 ± 0.14a
		N60	19.4 ± 0.05ad	33.3 ± 0.38a
		N80	19.1 ± 0.04be	28.3 ± 0.38a
		N100	19.9 ± 0.03ac	12.5 ± 0.22cf
		N120	2.0 ± 0.03j	17.5 ± 0.01bc
		N140	3.7 ± 0.05ij	3.3 ± 0.03h
LSD (p < 0.05)			0.48	0.6
Level of Significance				
Tillage x Residue			^***^	^*^
Fertilizer x Tillage			^***^	^***^
Fertilizer x Residue			ns	^***^
Fertilizer x Tillage x Residue			^***^	^**^

CT = conventional tillage; ST = strip tillage; HR = higher residue: 30 cm of rice and wheat residue + 100% mungbean residue, by height; LR = lower residue: 15 cm of rice and wheat residue + 100% mungbean residue; N0 = 0% of recommended dose; N60 = 60% of recommended dose; N80: 80% of recommended dose; N100: 100% of recommended dose; N120: 120% of recommended dose; N140: 140% of recommended dose.

ns,

*,

**,

***indicate significant difference at the level P > 0.05, P < 0.05, P < 0.01, P < 0.001, respectively. Means separated by same lower-case letter (a, b, and c) under each column were not significantly different at p > 0.05 among different treatment combinations.

Residue-P showed a broader range (3.3–34.2 mg kg^-1^) across all the treatment combinations and was significantly affected by the interaction of tillage, residue levels and N fertilizers rate (P < 0.01, Table 3). The highest content (34.2 mg kg^-1^) was found in ST-LR-N0, while the lowest (3.3 mg kg^-1^) observed under ST-LR-N140. Residue-P decreased sharply with increasing N rates, particularly under LR treatments and tillage treatments didn’t exhibit notable impacts on the residue-P fractions.

## Discussions

### Labile phosphorus fractions under different management practices

Labile phosphorus fractions, particularly soluble-P, NaHCO_3_-Pi (inorganic), and NaHCO_3_-Po (organic) are considered the most plant-available P forms [42] and act as the first responders to nutrient interventions. Our results showed that strip tillage (ST) combined with high residue (HR) retention significantly enhanced labile P fractions compared to conventional tillage (CT) with low residue (LR) system. This effect was especially pronounced under moderate nitrogen (N) application rates (≤N100), suggesting a synergistic relationship among reduced soil disturbance, organic matter input, and optimized N supply. Our results are also in line with Sharma et al. [43] who indicated that individual tillage treatments did not significantly alter the soluble P content. The positive effect of HR on soluble-P contents supports previous findings [5,44–46] which observed that surface-retained residues reduce P adsorption by mineral surfaces due to increased soil organic matter and competitive anion interactions. According to Hou et al. [47], soluble-P may rise as a result of dissolved soil organic matter or organic acids buildup, which mobilize P through ligand exchange, restrict P sorption and enhance P solubility in soil. Our results align with findings from Reddy et al. [6], Kumawat et al. [48] and Redel et al. [49], who observed increased labile P accumulation in surface soils under conservation agriculture (CA) practices. The improvement under ST-HR may be attributed to minimal soil disturbance, reduced fertilizer-soil contact and less P fixation and increased soil organic carbon (SOC) improving microbial activity and promoting mineralization of organic P (NaHCO_3_-Po) [45,50].

Higher N rates (≥N100) tended to decrease NaHCO_3_-Po levels across tillage and residue treatments, indicating a possible shift from organic to inorganic P pools or increased mineralization under high N loading, or likely due to microbial turnover and mineralization rates exceeding organic inputs. The ST-HR-N0 treatment yielded the maximum NaHCO_3_-Pi contents indicating that low N input under ST-HR conditions preserves labile Pi, possibly by limiting microbial immobilization or P uptake by crops. Under ST-LR and CT-LR, higher N reduced labile-Pi contents in soil. The drop in soil NaHCO_3_-Pi concentration may be attributed to the increased N levels which promoted plant development and root P uptake and was subsequently distributed to other plant parts [51]. This depletion could also reflect intensified biological uptake, microbial immobilization, or conversion into less available forms due to acidification and fixation, a phenomenon previously noted by Zhao et al. [51] and Bolo et al. [52]. Although higher N rates (N120 and N140) can increase total biomass and potentially P uptake, they reduce labile-P pools, suggesting a mismatch between nutrient input and plant/microbial demand. According to Hu et al. [53], higher N rate promotes potential net N mineralization and nitrification processes which may raise the biotic P requirement [54]. Higher N rates (>N100) reduced labile P levels, potentially due to acidification effects of ammonium-based fertilizers, enhanced plant uptake, or microbial immobilization of P [51,53]. Moreover, ammonium ions (NH_4_⁺) from urea fertilizers can interact with soil particles and increase adsorption of labile-P fractions due to electrostatic interactions. Past researchers have argued that the soluble-P may be decreased as a result of P fixation, leaching and low phosphate ion mobility [12,52], because higher soluble-P is the result of a synergistic impact between increased accessible Pi and increased N [44]. These findings underscore the importance of synchronizing nutrient application with biologically driven P cycling under CA and also imply that optimizing N rate under CA can maintain labile P while minimizing environmental risks like eutrophication, groundwater toxicity etc.

### Moderately labile phosphorus fractions under different management practices

Moderately labile P (NaOH-Pi and NaOH-Po) which is chemisorbed to Fe/Al oxides or complexed with humic substances, appears to act as a transitional pool between labile and stable forms, contributing to long-term P buffering [11,55]. Conservation agriculture can improve the moderately labile P fractions [45] which as evident in the present study. Strip tillage with high residue and elevated N rates (≥ N100) consistently enhanced moderately labile P pools (NaOH-Pi and NaOH-Po), indicating that these forms are strongly influenced by biologically mediated processes and soil chemical conditions. Moreover, our study showed that NaOH-P fractions were consistently higher under ST than CT, emphasizing the role of reduced disturbance in conserving organic inputs and promoting microbial processes. The concurrent increase in NaOH-Po suggests enhanced microbial biomass turnover and organic matter accumulation, especially under HR conditions.

High nitrate (NO_3_⁻) concentrations released from N fertilizers might acidify soils, enhancing desorption of Fe/Al-bound P and shifting equilibrium toward NaOH-Pi fractions [56,57]. Moreover, the reasons might be soil pH changes, microbial dynamics, ionic interactions, and redox conditions that are influenced by increased NO_3_ ⁻ –N concentrations. Higher NO_3_ ⁻ ions could enhance microbial activity and alter the adsorption-desorption equilibrium that convert organic phosphorus to inorganic forms. The findings of our study align with other researchers [56–59], who found that N-induced microbial activity facilitates the mobilization of moderately labile P pools. Higher NaOH-Pi pools with higher N rates in our study can be attributed to mobilization of NaOH-Pi fraction to augment the sufficient amount of accessible Pi for crop growth. The decline in NaOH-Pi pools indicated that the size of the NaHCO_3_-Pi pool in the soil might not be sufficient to meet crop P needs [60] and thus NaOH-Pi pools were converted to labile Pi fractions. The NaOH-Po pool may serve as the primary source of plant-available P in the soil and sustain plant-available P levels [61]. Lower mineralization would be expected at lower N supply resulting in lower NaOH-Po pools. In situations where soil P supply is low, Po mineralization serves as crops’ primary source of available Pi resulting in lower NaOH-Po pools [62]. Thus, adoption of optimum N rate is crucial for preventing loss of moderately labile-P pools through erosion, runoff or other factors.

This study also contributes novel insight into how management-driven shifts in microbial-mediated processes affect intermediate P pools. It also emphasizes the buffering role of NaOH-P fractions, suggesting that regulating N inputs with CA practices can sustain crop-available P over time while minimizing external P input requirements. Nevertheless, this study did not directly measure microbial biomass or enzyme activity, which limits our ability to conclusively link biological processes with P transformations. Future research should integrate microbial and biochemical indicators to strengthen the understanding.

### Non-labile phosphorus fractions under different management practices

The recalcitrant P pools (acid-P and residual-P) are generally considered stable, non-labile and unavailable for immediate plant uptake but represent important long-term nutrient reserves. The acid-P fraction contained P bonded to Ca [55] and the quantity is high in alkaline soil due to the high stability of Ca-P [44]. The levels of acid-P varied depending on the tillage and residue management coupled with N rate. In our study, Acid-P levels increased under ST and HR that was opposite to the findings of Haokip et al. [45] who found no significant interaction between tillage and nutrients on acid-P. Acid-P was higher under ST, particularly when coupled with lower N rate (≤N100) and HR. In soils near neutral pH (as in our site), Ca-bound P is not readily soluble; however, high N may contribute to acidification and enhance solubilization, as suggested by Jing et al. [63]. This may explain the decline in acid-P at higher N rates (N120, N140), possibly due to hydrolysis or transformation of a relatively resistant acid-P pool into labile or moderately labile forms under increased plant/microbial demand [63,64]. The acid-P fractions’ altered concentrations suggest that these fractions may be involved in long-term P cycling. Under CA, the acid-P fractions may function as a slow-release storage of P, ensuring that plants constantly have a supply of available P even when short-term inputs are altered, supporting long-term soil fertility and crop production. Since crop demand for P absorption could be increased by high N fertilizer, it is possible that acid-P depletion occurred in addition to labile Pi fractions [64]. A portion of the acid-P pool’s hydrolysis products would move straight into the NaHCO_3_-Pi pool. Nevertheless, the portions of Pi near the surface of the original mineral particles might easily be re-adsorbed and fixed again and merged into acid-P if the released P or the immediately generated NaHCO_3_-Pi cannot be utilized by crops or microorganisms in time; and it is also possible for other P pools to partially complement acid-P pools [63]. Through better soil, crop and nutrient management, these non-labile P pools can be utilized over the multiple cropping seasons and chemical P inputs can be minimized.

The residual-P pools are unlikely to provide short-term contributions to the soil solution and plant nutrients [65]. Residual-P, consisting of mineral-occluded and highly refractory forms, declined under high N rates, particularly under ST with LR. This pattern may reflect increased P demand, release of P from residual P pools and mobilization via microbial activity, as supported by findings of Fan et al. [46] and Mahmood et al. [58]. Lower residues might have boosted residual-P at lower N rates, whereas higher residues might have decreased its mobilization due to the residual-P’s persistence, resistance, and insoluble nature. The gradual decrease in residual P suggests that even recalcitrant pools can contribute to P cycling under prolonged cultivation and appropriate stimulation, reinforcing the importance of integrated nutrient management [65]. Interestingly, residual-P content declined with increasing N rate and HR indicating that residual-P can be biologically mobilized over time, especially under improved soil conditions and active root growth [66]. An increase in biomass among the plants (particularly root length and root hair density) during P shortage can enhance plant performance and greatly boost residual P absorption [66,67]. When agricultural residues are added to soil systems, the residual P portion could function as a sink [68]. The transformation of residual P into more available forms under CA practices and moderate N rates suggests that such systems can gradually tap into native soil reserves, reducing dependence on external P inputs. However, the variability in non-labile P response across treatments and the lack of significant trends for some interactions highlight the complexity of these pools and their resistance to short-term changes. It also suggests the need for longer monitoring or inclusion of isotopic P labeling techniques to track P transformations.

## Conclusions

This long-term field study demonstrates that phosphorus availability and transformation in intensively managed cropping system are significantly influenced by the interactive effects of tillage practices, crop residue retention, and nitrogen input levels. Specifically, strip tillage combined with high residue retention and moderate to higher nitrogen application enhanced labile and moderately labile phosphorus pools, while conventional tillage with lower residue incorporation under lower nitrogen rates tended to deplete these pools and increase phosphorus fixation into non-labile forms. A larger amount of non-labile phosphorus pool under both conventional and conservation agricultural practices was obtained with moderate nitrogen inputs, however, higher rates of nitrogen fertilization reduced this pool. These findings underscore the mechanistic link between soil tillage and nitrogen management and the maintenance of dynamic phosphorus pools that is critical for the sustenance of soil fertility and sustainable crop productivity. The novelty of this study lies in its comprehensive evaluation of phosphorus fractions under long-term, real-world agronomic practices, moving beyond total phosphorus to show how specific management choices alter available and stable phosphorus pools. The study contributes novel evidence on phosphorus dynamics under tropical conservation agriculture systems and provides valuable insights for refining site-specific efficient nutrient management strategies, particularly in intensive cereal-based cropping systems of South Asia. The findings provide a scientific basis for promoting conservation agriculture practices that enhance phosphorus use efficiency and contribute to long-term soil fertility. However, the study was conducted under specific agro-climatic and soil conditions, which may limit the direct applicability of results to other regions. Future studies should explore microbial and enzymatic processes associated with phosphorus cycling and assess crop uptake dynamics in conjunction with soil phosphorus fractions.

## Supporting information

S1 Table Supporting information_raw dataset. (XLSX)

## References

[1] Tang X, Li Z, Ma Y, Liang Y. Mechanism of fulvic acid-and organic manure-mediated phosphorus mobilization in black soil at low temperature. J Plant Nutri Fertilizers. 2012;18:893–9.

[2] Mayakaduwage S, Alamgir M, Mosley L, Marschner P. Phosphorus pools in sulfuric acid sulfate soils: influence of water content, pH increase and P addition. J Soils Sediments. 2019;20(3):1446–53. doi: 10.1007/s11368-019-02521-1

[3] Kumar U, Cheng M, Islam MJ, Maniruzzaman M, Nasreen SS, Haque ME, et al. Retention of Crop Residue Increases Crop Productivity and Maintains Positive Sulfur Balance in Intensive Rice-Based Cropping Systems. J Soil Sci Plant Nutr. 2024;24(4):6856–67. doi: 10.1007/s42729-024-02009-z

[4] Tiefenbacher A, Sandén T, Haslmayr HP, Miloczki J, Wenzel W, Spiegel H. Optimizing carbon sequestration in croplands: A synthesis. Agron J. 2021;11:882.

[5] Nunes R de S, de Sousa DMG, Goedert WJ, de Oliveira LEZ, Pavinato PS, Pinheiro TD. Distribution of Soil Phosphorus Fractions as a Function of Long-Term Soil Tillage and Phosphate Fertilization Management. Front Earth Sci. 2020;8. doi: 10.3389/feart.2020.00350

[6] Reddy D, Kushwah S, Srivastava S, Khamparia RS. Long-term wheat residue management and supplementary nutrient input effects on phosphorus fractions and adsorption behavior in a Vertisol. Communications Soil Sci Plant Analysis. 2014;45:541–54.

[7] Hu B, Yang B, Pang X, Bao W, Tian G. Responses of soil phosphorus fractions to gap size in a reforested spruce forest. Geoderma. 2016;279:61–9.

[8] Jindo K, Audette Y, Olivares FL, Canellas LP, Smith DS, Paul Voroney R. Biotic and abiotic effects of soil organic matter on the phytoavailable phosphorus in soils: a review. Chem Biol Technol Agric. 2023;10(1):29. doi: 10.1186/s40538-023-00401-y 37026154 PMC10069009

[9] Williams A, Börjesson G, Hedlund K. The effects of 55 years of different inorganic fertiliser regimes on soil properties and microbial community composition. Soil Biol Biochem. 2013;67:41–6.

[10] Meena AL, Pandey RN, Behera UK, Meena MD. Effect of tillage and crop residue management on phosphorus transformation and phosphatase enzyme activity under pigeonpea (Cajanus cajan) rhizosphere and non-rhizosphere soil. Annals Biol. 2013;29(2):148–57.

[11] Liu Y, Feng L, Hu H, Jiang G, Cai Z, Deng Y. Phosphorus release from low grade rock phosphates by low molecular weight organic acids. J Food Agric Environ. 2012;10:1001–7.

[12] Amaizah NR, Čakmak D, Saljnikov E, Roglić G, Mrvic V, Krgović R, et al. Fractionation of soil phosphorus in a long-term phosphate fertilization. J Serb Chem Soc. 2012;77(7):971–81.

[13] Duncan EG, O’Sullivan CA, Roper MM, Biggs JS, Peoples MB. Influence of co-application of nitrogen with phosphorus, potassium and sulphur on the apparent efficiency of nitrogen fertiliser use, grain yield and protein content of wheat: review. Field Crops Research. 2018;226:56–65.

[14] Zhu X, Chen H, Zhang W, Huang J, Fu S, Liu Z, et al. Effects of nitrogen addition on litter decomposition and nutrient release in two tropical plantations with N2-fixing vs. non-N2-fixing tree species. Plant Soil. 2016;399(1):1–14.

[15] Egashira K, Takenaka J, Shuto S, Moslehuddin AZM. Phosphorus status of some paddy soils in Bangladesh. Soil Sci Plant Nutr. 2003;49(5):751–7.

[16] Rahman MA, Didenko NO, Sundermeier AP, Islam KR. Agricultural management systems impact on soil phosphorous partition and stratification. Water Air Soil Pollut. 2021;232(6):1–15. doi: 10.1007/S11270-021-05196-Y

[17] Neugschwandtner RW, Liebhard P, Kaul HP, Wagentristl H. Soil chemical properties as affected by tillage and crop rotation in a long-term field experiment. Plant Soil Environ. 2014;60(2):57–62.

[18] Wei T, Zhang P, Wang K, Ding R, Yang B, Nie J, et al. Effects of wheat straw incorporation on the availability of soil nutrients and enzyme activities in semiarid areas. PLoS One. 2015;10(4):e0120994. doi: 10.1371/journal.pone.0120994 25880452 PMC4400158

[19] Saurabh K, Rao KK, Mishra JS, Kumar R, Poonia SP, Samal SK, et al. Influence of tillage based crop establishment and residue management practices on soil quality indices and yield sustainability in rice-wheat cropping system of Eastern Indo-Gangetic Plains. Soil Tillage Res. 2021;206:104841. doi: 10.1016/j.still.2020.104841 33536693 PMC7722508

[20] Holanda FSR, Mengel DB, Paula MB, Carvaho JG, Bertoni JC. Influence of crop rotations and tillage systems on phosphorus and potassium stratification and root distribution in the soil profile. 2008;29(15–16):2383–94.

[21] Baker DB, Johnson LT, Confesor RB, Crumrine JP. Vertical Stratification of Soil Phosphorus as a Concern for Dissolved Phosphorus Runoff in the Lake Erie Basin. J Environ Qual. 2017;46(6):1287–95. doi: 10.2134/jeq2016.09.0337 29293833

[22] Liu J, Macrae ML, Elliott JA, Baulch HM, Wilson HF, Kleinman PJA. Impacts of Cover Crops and Crop Residues on Phosphorus Losses in Cold Climates: A Review. J Environ Qual. 2019;48(4):850–68. doi: 10.2134/jeq2019.03.0119 31589697

[23] Chang SC, Jackson ML. Fractionation of soil phosphorus. Soil Sci. 1957;84:133–44.

[24] Hingston FJ, Atkinson RJ, Posner AM, Quirk JP. Specific adsorption of anions. Nature. 1967;215(5109):1459–61.

[25] Hedley MJ, Stewart JWB, Chauhan BS. Changes in inorganic and organic soil phosphorus fractions induced by cultivation practices and by laboratory incubations. Soil Sci Soc Am J. 1982;46:970–6.

[26] Tiessen H, Moir JO. Characterization of available P by sequential extraction. In: Carter MR, Gregorich EG, editors. Soil sampling and methods of analysis. 2 ed. Boca Raton, FL, USA: Canadian Society of Soil Science. 2008:293–306.

[27] Popović AR, Đorđević D. Speciation of selected trace and major elements in lignite used in ‘Nikola Tesla A’ power plant (Obrenovac, Serbia). J Serb Chem Soc. 2005;70(12):1497–513.

[28] Maniruzzaman, Cheng M, Islam A, Kumar U, Islam J, Nasreen SS, et al. Conservation agriculture increases phosphorus pools and stock in the top 30 cm of soil: A three‐year study on a subtropical legume–rice rotation. Soil Use and Management. 2024;40(4). doi: 10.1111/sum.13153

[29] Mehra P, Baker J, Sojka RE, Bolan N, Desbiolles J, Kirkham MB, et al. A review of tillage practices and their potential to impact the soil carbon dynamics. Adv Agron. 2018;150:185–230.

[30] Oladele SO, Adetunji AT. Agro-residue biochar and N fertilizer addition mitigates CO2-C emission and stabilized soil organic carbon pools in a rain-fed agricultural cropland. Int Soil Water Conser Res. 2021;9(1):76–86.

[31] Kumar A, Deori C, Divya D, Chauhan GV, Pradhan R, Kashyap M, et al. Conservation agriculture: effect on crop yield, soil health and carbon sequestration in Indian soils. Plant Archives. 2025;25(1):635–46.

[32] Margenot AJ, Paul BK, Sommer RR, Pul-leman MM, Parikh SJ, Jackson LE, et al. Can conservation agriculture improve phosphorus (P) availability in weathered soils? Effects of tillage and residue management on soil P status after 9 years in a Kenyan Oxisol. Soil and Tillage Research. 2017;166:157–66.

[33] Palm C, Blanco-Canqui H, De C lerck F, Gatere L, Grace P. Conservation agriculture and ecosystem services: An over-view. Agric Ecosys Environ. 2014;187:87–105.

[34] Nannipieri P, Giagnoni L, Landi L, Renella G. Role of phosphatase enzymes in soil. In: Bünemann E, Oberson A, Frossard E, editors. Phosphorus in action. Berlin, Heidelberg: Springer. 2011.

[35] Baan CD, Grevers MCJ, Schoenau JJ. Effects of a single cycle of tillage on long-term notill prairie soils. Canadian Journal of Soil Science. 2009;89:521–30.

[36] Carreira JA, García-Ruiz R, Liétor J, Harrison AF. Changes in soil phosphatase activity and P transformation rates induced by application of N- and S-containing acid-mist to a forest canopy. Soil Biol Biochem. 2000;32:1357–865.

[37] Klammsteiner T, Turan V, Fernández-Delgado Juárez M, Oberegger S, Insam H. Suitability of Black Soldier Fly Frass as Soil Amendment and Implication for Organic Waste Hygienization. Agronomy. 2020;10(10):1578. doi: 10.3390/agronomy10101578

[38] FRG. Fertilizer Recommendation Guide. Dhaka: Bangladesh Agricultural Research Council. 2018.

[39] Saleque MA, Naher UA, Islam A, Pathan ABMBU, Hossain ATMS, Meisner CA. Inorganic and organic phosphorus fertilizer effects on the phosphorus fractionation in wetland rice soils. Soil Sci Soc Am J. 2004;68(5):1635–44.

[40] Murphy J, Riley JP. A modified single solution method for the determination of phosphate in natural waters. Analytica Chimica Acta. 1962;27:31–6. doi: 10.1016/s0003-2670(00)88444-5

[41] Biswas C, Ferdous J, Sarker RR, Islam KR, Jahangir MMR. Forty-two years impact of chemical fertilization on soil phosphorus partition and distribution under rice-based cropping systems. PLoS One. 2024;19(6):e0305097. doi: 10.1371/journal.pone.0305097 38857213 PMC11164328

[42] Blake L, Johnston AE, Poulton PR, Goulding KWT. Changes in soil phosphorus fractions following positive and negative phosphorus balances for long periods. Plant Soil. 2003;254:245–61.

[43] Sharma KL, Indoria AK, Srinivas K, Reddy KS, Charry GR, Lal M, et al. Effect of long-term use of tillage, crop residue and N application on phosphorus fractions in soil under sorghum (Sorghum vulgare L.)-castor System (Ricinus communis) in Rainfed Alfisol Soils. Commun Soil Sci Plant Analys. 2020;51(1):1–13.

[44] Anil AS, Sharma VK, Jiménez-Ballesta R, Parihar CM, Datta SP, Barman M, et al. Impact of long-term conservation agriculture practices on phosphorus dynamics under maize-based cropping systems in a sub-tropical soil. Land. 2022;11:1488.

[45] Haokip IC, Dwivedi BS, Meena MC, Datta SP, Jat HS, Dey A, et al. Effect of conservation agriculture and nutrient management options on soil phosphorus fractions under maize-wheat cropping system. J Indian Soc Soil Sci. 2020;68(1):45. doi: 10.5958/0974-0228.2020.00005.5

[46] Fan Y, Lin F, Yang L, Zhong X, Wang M, Zhou J, et al. Decreased soil organic P fraction associated with ectomycorrhizal fungal activity to meet increased P demand under N application in a subtropical forest ecosystem. Biol Fertil Soils. 2017;54(1):149–61. doi: 10.1007/s00374-017-1251-8

[47] Hou E, Tang S, Chen C, Kuang Y, Lu X, Heenan M, et al. Solubility of phosphorus in subtropical forest soils as influenced by low-molecular organic acids and key soil properties. Geoderma. 2018;313:172–80.

[48] Kumawat C, Sharma VK, Meena MC, Dwivedi BS, Barman M, Kumar S, et al. Effect of crop residue retention and phosphorus fertilization on P use efficiency of maize and biological properties of soil under maize-wheat cropping system in an Inceptisol. Indian J Agric Sci. 2018;88:1184–9.

[49] Redel YD, Rubio R, Rouanet JL, Borie F. Phosphorus bioavailability affected by tillage and crop rotation on a Chilean volcanic derived Ultisol. Geoderma. 2007;139:388–96.

[50] Sharma S, Kaur S, Parkash Choudhary O, Singh M, Al-Huqail AA, Ali HM, et al. Tillage, green manure and residue retention improves aggregate-associated phosphorus fractions under rice-wheat cropping. Sci Rep. 2022;12(1):7167. doi: 10.1038/s41598-022-11106-x 35504974 PMC9064998

[51] Zhao Q, Liu XY, Hu YL, Zeng DH. Effects of nitrogen addition on nutrient allocation and nutrient resorption efficiency in Larix gmelinii. Scientia Silvae Sinica. 2010;46:14–9.

[52] Bolo P, Kihara J, Mucheru-Muna M, Njeru EM, Kinyua M, Sommer R. Application of residue, inorganic fertilizer and lime affect phosphorus solubilizing microorganisms and microbial biomass under different tillage and cropping systems in a Ferralsol. Geoderma. 2021;390:114962.

[53] Hu YL, Zeng DH, Liu YX, Zhang YL, Chen ZH, Wang ZQ. Responses of soil chemical and biological properties to nitrogen addition in a Dahurian larch plantation in Northeast China. Plant Soil. 2010;333:81–92.

[54] Kader MA, Jahangir MMR, Islam M, Begum R. Long-term conservation agriculture increases nitrogen use efficiency by crops, land equivalent ratio and soil carbon stock in a subtropical rice-based cropping system. Field Crops Research. 2022;287:108636.

[55] Cross AF, Schlesinger WH. A literature review and evaluation of the Hedley fractionation: applications to the biogeochemical cycle of soil phosphorus in natural ecosystems. Geoderma. 1995;64:197–214.

[56] Qaswar M, Jing H, Ahmed W, Abbas M, Dongchu L. Linkages between ecoenzymatic stoichiometry and microbial community structure under long-term fertilization in paddy soil: a case study in China. Applied Soil Ecology. 2021;161:103860.

[57] Chen H, Chen M, Li D, Mao Q, Zhang W, Mo J. Responses of soil phosphorus availability to nitrogen addition in a legume and a non-legume plantation. Geoderma. 2018;322:12–8.

[58] Mahmood M, Tian Y, Ma Q, Hui X, Elrys AS, Ahmed W, et al. Changes in phosphorus fractions in response to long-term nitrogen fertilization in loess plateau of China. Field Crops Research. 2021;270.

[59] Yang K, Zhu J, Gu J, Yu L, Wang Z. Changes in soil phosphorus fractions after 9 years of continuous nitrogen addition in a Larix gmelinii plantation. Annals Forest Sci. 2015; 72(4): 435–42. doi: 10.1007/s13595-014-0444-7

[60] Hassan HM, Marschner P, McNeill A, Tang C. Grain legume pre-crops and their residues affect the growth, P uptake and content of P pools in the rhizosphere of the following wheat. Biol Fert Soils. 2012;48:775–85.

[61] Beck MA, Sanchez PA. Soil phosphorus fraction dynamics during 18 years of cultivation on a Typic Paleudult. Soil Sci Soc Am J. 1994;58:1424–31.

[62] Liao D, Zhang C, Li H, Lambers H, Zhang F. Changes in soil phosphorus fractions following sole cropped and intercropped maize and faba bean grown on calcareous soil. Plant Soil. 2020;448:587–601. doi: 10.1007/s11104-020-04460-0

[63] Jing D, Yan Y, Ren T, Lu J, Wang X, Tan W, et al. Effects of nitrogen application rate on phosphorus transformation in an Alfisol: Results from phosphate-oxygen isotope ratios. Applied Geochem. 2021;134:105094.

[64] Sun W, Villamil MB, Behnke GD, Margenot AJ. Long-term effects of crop rotation and nitrogen fertilization on phosphorus cycling and balances in loess-derived Mollisols. Geoderma. 2022;420.

[65] Shi Y, Ziadi N, Messiga AJ, Lalande R, Hu Z. Changes in soil phosphorus fractions for a long‐term corn‐soybean rotation with tillage and phosphorus fertilization. Soil Sci Soc Am J. 2013;77(4):1402–12.

[66] McDowell RW, Condron LM, Stewart I. Variation in environmentally- and agronomically-significant soil phosphorus concentrations with time since stopping the application of phosphorus fertilisers. Geoderma. 2016;280:67–72.

[67] Irfan M, Aziz T, Maqsood MA, Bilal HM, Siddique KHM, Xu M. Phosphorus (P) use efficiency in rice is linked to tissue-specific biomass and P allocation patterns. Sci Rep. 2020;10(1):4278. doi: 10.1038/s41598-020-61147-3 32152340 PMC7062884

[68] Hao X, Godlinski F, Chang C. Distribution of phosphorus forms in soil following long term continuous and discontinuous cattle manure applications. Soil Sci Soc Am J. 2008;72:90–7.

