# Supplementary material for: Long-term conservation agriculture with optimum nitrogen fertilization improves soil phosphorus availability
Source: PLoS One. 2025 Sep 24;20(9):e0333013. doi: 10.1371/journal.pone.0333013 (PMC12459793; doi:10.1371/journal.pone.0333013)

PLOS ONE

Effect of long-term conservation agriculture with nitrogen rates on soil available and labile phosphorus pools

--Manuscript Draft--

Manuscript Number:	PONE-D-25-17789
Article Type:	Research Article
Full Title:	Effect of long-term conservation agriculture with nitrogen rates on soil available and labile phosphorus pools
Short Title:	Soil management's influence on soil phosphorus fractions
Corresponding Author:	Nusrat Jahan Mumu<br>khulna Agricultural University<br>khulna, BANGLADESH
Keywords:	Phosphorus fractions; labile P; non-labile P; conservation agriculture; residue retention
Abstract:	Conservation agriculture (CA) practices influence soil phosphorus (P) dynamics and pools by altering phosphorus stoichiometry. However, the dynamics of P pools and their availability under long-term CA practices are not extensively understood. This study aimed to evaluate the effects of different nitrogen (N) rates with long-term CA practices on different soil P fractions and availability under wheat-mungbean-rice cropping systems. Labile, moderately labile, and non-labile P fractions were measured after 36 crop cycles under two tillage methods (conventional tillage, CT; strip tillage, ST), two residue retention levels (lower residue (LR); higher residue (HR)), and six nitrogen (N) rates (N0: 0%; N1: 60%; N2: 80%; N3: 100%; N4: 120%; N5: 140% of the recommended dose). Higher residue (HR) retention improved labile P pools, suggesting its potential role in enhancing soil fertility under CA practice. The results indicated significantly higher soluble-P in the CT-HR-N3 treatment. The $NaHCO_3$-P (inorganic) was higher in HR coupled with lower N rate (≤ N3). Recommended N rate under CA aids in raising the $NaHCO_3$-P (organic) fractions. Moderately labile-P fractions were higher in ST-HR coupled with higher N rate (> N3). Acid-P was consistently higher in ST-HR couple with lower N rate (≤ N3) while the residual-P was higher in ST-LR coupled with lower N rate. Loosely bound-Pi pools were observed with a decreasing trend with higher N rates. The results indicate that crop residue retention enhances most soil P fractions, while higher chemical N rates reduce them. Estimation of a suitable N rate under CA practices can help sustain plant available P while balancing P dynamics between non-labile and moderately labile pools. In the study, N3 was found the most effective rate and combination of ST-HR-N3 can be the best option for sustaining P availability while maintaining soil health.
Order of Authors:	Nusrat Jahan Mumu
	Sunjana Akter
	Jannatul Ferdous
	Nushaiba Atiq Taima
	Most. Khatiza Khatun
	Md. Mofizur Rahman Jahangir
Additional Information:	
Question	Response
**Financial Disclosure**<br><br>Enter a financial disclosure statement that describes the sources of funding for the work included in this submission. Review the submission guidelines for detailed	The author(s) received no specific funding for this work.

Powered by Editorial Manager® and ProduXion Manager® from Aries Systems Corporation

requirements. View published research articles from *PLOS ONE* for specific examples.<br><br>This statement is required for submission and **will appear in the published article** if the submission is accepted. Please make sure it is accurate.<br><br>**Funded studies**<br>Enter a statement with the following details:<br>• Initials of the authors who received each award<br>• Grant numbers awarded to each author<br>• The full name of each funder<br>• URL of each funder website<br>• Did the sponsors or funders play any role in the study design, data collection and analysis, decision to publish, or preparation of the manuscript?<br><br>Did you receive funding for this work?	
**Competing Interests**<br><br>Use the instructions below to enter a competing interest statement for this submission. On behalf of all authors, disclose any competing interests that could be perceived to bias this work—acknowledging all financial support and any other relevant financial or non-financial competing interests.<br><br>This statement is required for submission and **will appear in the published article** if the submission is accepted. Please make sure it is accurate and that any funding sources listed in your Funding Information later in the submission form are also declared in your Financial Disclosure statement.<br><br>View published research articles from *PLOS ONE* for specific examples.	The authors have declared that no competing interests exist.

**NO authors have competing interests**<br><br>Enter: *The authors have declared that no competing interests exist.*<br><br>**Authors with competing interests**<br><br>Enter competing interest details beginning with this statement:<br><br>*I have read the journal's policy and the authors of this manuscript have the following competing interests: [insert competing interests here]*<br><br>* typeset	
**Ethics Statement**<br><br>Enter an ethics statement for this submission. This statement is required if the study involved:<br><br>• Human participants<br>• Human specimens or tissue<br>• Vertebrate animals or cephalopods<br>• Vertebrate embryos or tissues<br>• Field research<br><br>Write "N/A" if the submission does not require an ethics statement.<br><br>General guidance is provided below. Consult the submission guidelines for detailed instructions. **Make sure that all information entered here is included in the Methods section of the manuscript.**	Field research was conducted in the Soil Science Field Laboratory at Bangladesh Agricultural University.

Powered by Editorial Manager® and ProduXion Manager® from Aries Systems Corporation

**Format for specific study types**<br><br>**Human Subject Research (involving human participants and/or tissue)**<br>• Give the name of the institutional review board or ethics committee that approved the study<br>• Include the approval number and/or a statement indicating approval of this research<br>• Indicate the form of consent obtained (written/oral) or the reason that consent was not obtained (e.g. the data were analyzed anonymously)<br><br>**Animal Research (involving vertebrate animals, embryos or tissues)**<br>• Provide the name of the Institutional Animal Care and Use Committee (IACUC) or other relevant ethics board that reviewed the study protocol, and indicate whether they approved this research or granted a formal waiver of ethical approval<br>• Include an approval number if one was obtained<br>• If the study involved *non-human primates*, add *additional details* about animal welfare and steps taken to ameliorate suffering<br>• If anesthesia, euthanasia, or any kind of animal sacrifice is part of the study, include briefly which substances and/or methods were applied<br><br>**Field Research**<br><br>Include the following details if this study involves the collection of plant, animal, or other materials from a natural setting:<br>• Field permit number<br>• Name of the institution or relevant body that granted permission	
**Data Availability**<br><br>Authors are required to make all data underlying the findings described fully available, without restriction, and from the time of publication. PLOS allows rare exceptions to address legal and ethical concerns. See the PLOS Data Policy and FAQ for detailed information.	No - some restrictions will apply

A Data Availability Statement describing where the data can be found is required at submission. Your answers to this question constitute the Data Availability Statement and **will be published in the article**, if accepted.<br><br>**Important:** Stating 'data available on request from the author' is not sufficient. If your data are only available upon request, select 'No' for the first question and explain your exceptional situation in the text box.<br><br>Do the authors confirm that all data underlying the findings described in their manuscript are fully available without restriction?	
**Describe where the data may be found in full sentences. If you are copying our sample text, replace any instances of XXX with the appropriate details.**<br><br>• If the data are **held or will be held in a public repository**, include URLs, accession numbers or DOIs. If this information will only be available after acceptance, indicate this by ticking the box below. For example: *All XXX files are available from the XXX database (accession number(s) XXX, XXX.).*<br>• If the data are all contained **within the manuscript and/or Supporting Information files**, enter the following: *All relevant data are within the manuscript and its Supporting Information files.*<br>• If neither of these applies but you are able to provide **details of access elsewhere**, with or without limitations, please do so. For example:<br><br>*Data cannot be shared publicly because of [XXX]. Data are available from the XXX Institutional Data Access / Ethics Committee (contact via XXX) for researchers who meet the criteria for access to confidential data.*<br><br>*The data underlying the results presented in the study are available from (include the name of the third party*	Data will be available upon request.

Powered by Editorial Manager® and ProduXion Manager® from Aries Systems Corporation

and contact information or URL).<br>• This text is appropriate if the data are owned by a third party and authors do not have permission to share the data.<br><br>* typeset	
Additional data availability information:	Tick here if the URLs/accession numbers/DOIs will be available only after acceptance of the manuscript for publication so that we can ensure their inclusion before publication.

Powered by Editorial Manager® and ProduXion Manager® from Aries Systems Corporation

Manuscript

Click here to access/download;Manuscript;3. Manuscript-NJ Mumu.docx

Effect of long-term cConservation tillage with nitrogen fertilization improves soil phosphorus availability and labile phosphorus pools

Nusrat Jahan Mumu[1,2,*], Sunjana Akter[2], Jannatul Ferdous[2], Nushaiba Atiq Taima[2], Most. Khatiza Khatun[2], Md. Mofizur Rahman Jahangir[2]

[1]Department of Soil Science, Khulna Agricultural University, Khulna-9100, Bangladesh

[2]Department of Soil Science, Bangladesh Agricultural University, Mymensingh-2202, Bangladesh

*Corresponding author:

nusratmumu43044@bau.edu.bd

Abstract

Conservation agriculture (CA) especially minimum tillage ~~practices~~ influence soil phosphorus (P) dynamics; ~~and pools by altering phosphorus stoichiometry.~~ however, the information on the dynamics of P pools and their availability under long-term CA practices is limited ~~are not extensively understood~~. This study aimed to evaluate the effects of variable tillage and different nitrogen (N) fertilization rates ~~with long-term CA practices~~ on different soil P fractions and availability under wheat ()-mungbean()-rice() cropping systems. Labile, moderately labile, and non-labile P fractions were measured after 36 crop cycles under two tillage methods (conventional tillage, CT; strip tillage, ST), two residue retention levels (lower residue (LR); higher residue (HR)), and six nitrogen (N) rates (N0: 0%; N1: 60%; N2: 80%; N3: 100%; N4: 120%; N5: 140% of the recommended dose). ~~Higher residue (HR) retention improved labile P pools, suggesting its potential role in enhancing soil fertility under CA practice. The~~ Results indicated significantly higher soluble-P in the CT-HR-N3 treatment. The $NaHCO_3$-P (inorganic) was higher in HR coupled with lower N rate (≤ N3). Recommended N rate under CA aids in raising the $NaHCO_3$-P (organic) fractions. Moderately labile-P fractions were higher in ST-HR coupled with higher N rate (≥ N3). Acid-P was consistently higher in ST-HR couple with lower N rate (≤ N3) while the residual-P was higher in ST-LR coupled with lower N rate. Loosely bound-Pi pools were observed with a decreasing trend with higher N rates. The results indicate that crop residue retention enhances most soil P fractions, while higher chemical N rates reduce them. Estimation of a suitable N rate under CA practices can help sustain plant available P while balancing P dynamics between non-labile and moderately labile pools. In the study, N3 was found the most effective rate and combination of ST-HR-N3 can be the best option for sustaining P availability while maintaining soil health.

Key words: Phosphorus fractions, labile P, non-labile P, conservation agriculture, residue retention.

Introduction

Phosphorus is an essential nutrient to support crop production. There are two primary forms of phosphorus (P) found in soils: organic P (Po) and inorganic P (Pi), which can be further classified as labile (solution P, $NaHCO_3$-Pi, and $NaHCO_3$-Po), moderately-labile (NaOH-Pi, NaOH-Po), and non-labile (acid P and residue P) P fractions (1. Hu et al., 2016). The soil P fractions (both Po and Pi) determine the amount of P availability for plants (2. Liu et al., 2012; 3. Williams et al., 2013). The equilibrium between labile and moderately labile P is faster than the equilibrium between labile and non-labile P (4. Meena et al., 2013). While geological transformations often dictate the long-term state of P, metabolic mechanisms that impact organic matter also have a significant impact on its short-term availability to crops. The concentration of P in the soil solution remains extremely low because of its strong tendency to be fixed in soils. Over 90% of the total P in soils exists in insoluble and fixed forms (5. Amaizah et al., 2012). Adequate P supply to plants is a function of P fixation, solubility, lability and build up in soils. Phosphorus availability may decline dramatically due to excessive and unsuitable long-term agronomic practices such as monocropping, improper crop rotations, over reliance on chemical fertilizers, excessive tillage, lack of organic amendments, improper irrigation management etc. (6. Tang et al., 2012). Thus, phosphorus decline can be minimized by shifting towards more sustainable agricultural practices that include diversified crop rotation, conservation tillage, organic matter replenishment, and appropriate nutrient management.

Conservation agriculture (CA) is becoming more interesting and popular worldwide because of its potential to maintain soil and maximize crop productivity (7. Andersson and Giller, 2012; 8. Powlson et al., 2016). Three fundamental concepts of CA set it apart from the conventional system: minimizing soil disturbance (minimum or no-tillage techniques), preserving an organic soil cover i.e., residue, and adding short-duration legumes to the crop rotation to diversify the system (9. Kassam et al., 2009). Residue incorporation provides a resilient soil cover and naturally increasing soil organic matter content in surface horizons (10. Tiefenbacher et al., 2021). Reduced tillage and residue retention have been shown to improve crop yields by increasing soil moisture, reducing soil erosion and runoff, and promoting the accumulation of soil organic matter (11. Brouder and Gomez-Macpherson, 2014; 12. Palm et al., 2014). Short-duration leguminous crops in cropping sequence improves soil health by enhancing nitrogen fixation, phosphorus cycling, adding organic matter, improving soil structure, and supporting microbial community, all of which contribute to a more sustainable farming system. Prolonged application of organic amendments or residue can raise microbial activity as well as the labile and moderately labile P fractions in soil, which enhances the likelihood that soil organic matter may mineralize. Usually, P fixation lowers the labile P concentration in the soil by increasing the interaction between fertilizer P and soil components. Crop residue incorporation in combination with reduced tillage may lessen soil P fixation, increase labile P content, and promote the development of organic P pools. Addition of P from supplemented residue can control P mineralization or immobilization in soil, which may change the dynamics of P pools and influences its availability (13. Singh et al., 2009; 14. Reddy et al., 2014; 15. Kumawat et al., 2018; 16. Kumar et al., 2018; 17. Haokip et al., 2019). Reduced tillage practices, crop rotation, and residue

management all have a combined impact on the dynamics of soil P in the soil profile, which partially reflects shifts in the Po pools (18. Bunemann et al., 2006).

According to 19. Zhu et al (2016), there are interrelated biogeochemical interactions between N and P. Microbial activity can affect the mineralization of Po and Pi fractions because of N application rates (20. Mayakaduwage et al., 2020). Nitrogenous fertilizers alter microbial activity and thereby P sorption on the soil surface, turning labile P into recalcitrant or moderately labile P (21. Carreira et al., 1990). Soil N input may raise Po content and plant demand for Pi by influencing primary production. Absorption and utilization of P are typically altered by soil conditions that also influence P fixation and its availability for plant uptake. Availability of soil P decreases with the addition of ammonium-based fertilizers like urea that lower soil pH and increase P fixation. The condition is made worse by higher rate of urea application (22. Duncan et al., 2018). Therefore, selecting the suitable N fertilizer and dose is necessary for the sustainable management of P in soils. Over use of chemical fertilizers with conservation agriculture can cause negative environmental impacts. Assessing whether N fertilization causes P limitation or availability and investigating the underlying mechanisms are also crucial to standardize location-specific P management strategies.

Phosphorus deficiency affects approximately 5.7 billion hectares of agricultural land worldwide (23. Dhillon et al., 2017). Total P concentration of the Bangladesh rice cultivating soils ranged from 172 to 604 mg kg^{-1} and in the Old Himalayan Piedmont Plain (AEZ-9), organic P fractions were extraordinarily higher than inorganic P fractions which significantly influenced the availability of P to plants (24. Egashira et al., 2003). An understanding of the long-term dynamics and availability of soil P can be determined

through analyzing differences in soil P fractions. 25. Chang and Jackson (1957) and 26. Hingston et al. (1967) established distinct sequential P fractionation processes to separate their different fractions for characterizing soil P in detail. Thereafter, the procedures of the fractionation were modified also (27. Popović and Đorđević, 2005). In most of the procedures, the first extraction step aims at removing the loosely bound P and exchangeable Ca^{2+}, possibly interfering with the subsequent extraction steps. The use of extractants in the second extraction step is based on the ability of ions to form a stable complex with Al and to extract selectively Al-bound P (27. Popović and Đorđević, 2005). 28. Hedley et al. (1982) developed a method suitable for P fractionations in calcareous soils. The most popular techniques for the sequential fractionation of P in soils are the Hedley method (Hedley et al., 1982) and its modification by 29. Tiessen and Moir (2008) that allow the characterization of various inorganic P and organic P fractions according to their solubility.

There is little data on how variations in tillage and residue management over years, along with varying amounts of N fertilizer in soil affect the P fractions, dynamics, and availability in Bangladesh specially where cereal crops are cultivated over the years. It was anticipated that proper CA practices (reduced tillage and high residue retention) along with optimum i.e., recommended N rate would increase plant-available Pi, decrease soil P fixation, boost Po storage in soil. The objectives of this study were (i) to evaluate the dynamics and fluctuations in soil P fractions under long-term CA techniques in combination with various N rates, (ii) to determine the optimum rate of N fertilizer for minimizing P fixation in the soil, considering the availability of labile P fractions in soil.

Materials and Methods

Site description

The research was conducted In the Soil Science Field Laboratory (24°71.60'N, 90°42.51'E) at Bangladesh Agricultural University,Mymensingh in Bangladesh. The field site belongs to agro-ecological zone-9, comprising Old Brahmaputra Floodplain soil, and is classified as non-calcareous dark gray floodplain soil under the Sonatala series. It has a silt loam in texture, is neutral in pH (6.5), and moderately drained. With an average yearly temperature of 26° C, 1800 mm of rainfall, and 85% relative humidity, the area experiences sub-tropical monsoon weather (Weather station, BAU). Rice and wheat are major cereal crops in Bangladesh. Farmers in Mymensingh region mainly follow wheat-fallow-T. aman rice or wheat-jute-T. aman rice cropping pattern in their arable lands. But in the experiment, we included mungbean which is a short duration leguminous crop as a part of conservation agricultural practice. Thus, the experiment followed wheat-mungbean-rice, an improved cropping pattern, repeated at this location for 12 years.

Experimental design and treatment applications

In the experimental site, conservation agriculture (minimum tillage, soil cover residue, and adding short-duration legumes to crop rotation) was implemented as a long-term practice since 2011. There were three sets of treatments in the split-split plot design of the experiment: 1) conventional tillage (CT) and strip tillage (ST- only the rows were tilled and the areas in between the rows were left untilled, tilled rows were used for planting crops) were assigned to the main plots; 2) crop residue retention was assigned to the sub-plots (higher residue, HR: 30 cm of rice and wheat residue + 100% mungbean residue, by height; lower residue, LR: 15 cm of rice and wheat residue + 100% mungbean

residue); and 3) N fertilizer dose: N0: 0% RD; N1: 60% RD; N2: 80% RD; N3: 100% RD; N4: 120% RD; N5: 140% RD were assigned to sub-sub plots with three replications. Thus, total number of plots was 72, including two tillage × two residue levels × six N rates × three replications. Recommended fertilizer dose was followed from the national fertilizer recommendations for the test crops (30. FRG, 2018). The 100% RD for wheat, mung bean and rice were 100, 20 and 120 kg N/ha, respectively. Each sub-sub plot was 13 m × 3 m and had a dividing bund between each plot. Urea, triple super phosphate, muriate of potash, gypsum, magnesium sulphate, zinc sulphate and boric acid were applied as source of N, P, K, S, Mg, Zn and B, respectively, following the national fertilizer recommendation dose according to specific crop requirement. During the final land preparation, a basal dose of every fertilizer had been put to every plot, with the exception of urea during rice and wheat cultivation. Urea was applied to each plot in three equal split applications in wheat and rice. Moreover, intercultural practices including irrigation, weeding, insect and disease management were done aligned with the cultural practices for Bangladesh agroecological zone-9.

Soil samples collection, processing and analysis

Composite soil samples were collected from each plot after thirty-six consecutive crop cycles (12 years following the repeated cropping sequence and conservation agriculture) in 2023. Soil samples were collected from 0-15 soil depth using an auger and a core sampler from five different spots per plot randomly after crop harvesting. The collected soil samples were intermixed thoroughly to make a composite soil samples, air-dried at room temperature well under the shade, passed through 2 mm sieve, and stored until laboratory analysis. One gram of finely grounded soil sample (0.2 mm) was extracted sequentially for soil P fractionations (Figure. 1) dully following modified

Hedley Method (Hedley et al., 1982) as described by 29. Tiessen and Moir (2008), with a further modification including the final soil digestion by nitric acid and perchloric acid (30. Saleque et al., 2004). The sequential extractions of seven P fractions including solution P, $NaHCO_3$-P (Pi and Po), NaOH-P (Pi and Po), acid P and residual P were done.

Figure 1. Different soil phosphorus fractions according to lability.

Solution P was extracted by 0.01 M $CaCl_2$ for inorganic P fraction. $NaHCO_3$-P fraction was extracted by 0.5 M $NaHCO_3$ and $NaHCO_3$-Po (organic) were estimated by deducting $NaHCO_3$-Pi (inorganic) from the total $NaHCO_3$-extractable P following H_2SO_4 and H_2O_2 digestion. NaOH-P fraction was extracted by 0.1 M NaOH. After being digested with H_2SO_4 and H_2O_2, NaOH-Pi was deducted from the total NaOH-extractable P to determine NaOH-Po. Acid-P was extracted by 0.5 M H_2SO_4 and residual-P was extracted by HNO_3 and $HClO_4$ (5:2 ratio). The absorbance of P was determined using a spectrophotometer at 882 nm wavelength.

Statistical analysis

Tillage, residues, and nitrogen rate were the fixed factors in a split-split plot (three-way analysis) analysis of variance (ANOVA). To determine the impact and interaction between and among tillage, residue, and N rate treatments, data was statistically analyzed using the Jamovi 1.0.0.0. (R Package) software. The comparison of means was tested by the Duncan's Multiple Range Test (DMRT) and least significant difference (LSD) at the $p < 0.05$ level using SPSS (IBM SPSS Statistics, version 20, IBM Corp).

Results

Effects of management practices on labile phosphorus fractions

The mean soluble P fractions in soils ranged from 1.0 to 13.4 mgkg^{-1}. While tillage treatments had no significant impact on soluble P, significant interaction effects were observed among tillage, residues, and N levels (Table 1). The higher soluble-P content was observed in the CT-HR-N3 treatment, while the lowest content was recorded in the CT-LR-N4 treatment (Table 2). Soluble P fraction decreased with the increasing N levels with either tillage and residue level (Table 2).

Table 1. Effects of long-term combined application of different tillage, residue and N application treatments on labile phosphorus fractions in wheat-mungbean-rice cropping system.

**Treatments**	**Labile phosphorus fractions**		
**Tillage**	**Soluble-P**	**$NaHCO_3$-Pi**	**$NaHCO_3$-Po**
CT	4.9±0.06ans	22.1±0.26a***	16.3±0.08ans
ST	5.1±0.06a	15.2±0.22b	22.0±0.14a
Statistics	ns	***	ns
**Residue**			
HR	7.9±0.05x***	28.0±0.23a***	21.4±0.14a*
LR	2.2±0.02y	9.3±0.12b	16.9±0.10b
Statistics	***	***	*
**Fertilizer**			
N0	3.6±0.03E***	34.5±0.43a***	17.6±0.10b*
N1 (…. kg N/ha	5.5±0.10bC	26.5±0.36b	19.9±0.23b
N2	5.6±0.11B	15.6±0.38c	18.9±0.14b
N3	6.0±0.15A	14.4±0.38d	23.3±0.28a
N4	5.3±0.13C	11.3±0.26e	17.3±0.24b
N5	4.1±0.09D	9.6±0.23f	18.1±0.23b
Statistics	***	***	*
Tillage x Residue	**	***	*
Fertilizer x Tillage	***	***	**

Fertilizer x Residue	***	***	***
Fertilizer x Tillage x Residue	***	***	***

CT= conventional tillage; ST= strip tillage; HR= higher residue: 30 cm of rice and wheat residue + 100% mungbean residue, by height; LR= lower residue: 15 cm of rice and wheat residue + 100% mungbean residue; N0= 0% of recommended dose; N1= 60% of recommended dose; N2: 80% of recommended dose; N3: 100% of recommended dose; N4: 120% of recommended dose; N5: 140% of recommended dose.

*, **, and ***Mean values separated by same lower-case letter (a vs. b), (x vs. y), or same upper-case letter (A, B, C, D, E, and F) in each column were not significantly different in response to tillage, residue amount, and N fertilization, respectively.

ns, *, **, *** indicate significant difference at the level P > 0.05, P < 0.05, P < 0.01, P < 0.001, respectively

Table 2. Labile-P fractions in soil (depth: 0-15cm) under long term conventional (CT) and strip (ST) tillage systems, high residue (HR) and low residue (LR) retention and six N application rates.

**Treatments**			**Labile Phosphorus Fractions**		
**Tillage**	**Residue**	**Fertilizer**	**Soluble-P (mgkg^{-1})**	**$NaHCO_3$-Pi (mgkg^{-1})**	**$NaHCO_3$-Po (mgkg^{-1})**
	HR	N0	3.3± 0.01g	44.9±0.25b	18.2± 0.45bd
		N1	4.3± 0.01f	43.2±0.02c	13.0± 0.45ce
		N2	5.5± 0.01e	35.9±0.01d	15.6± 0.78be
		N3	13.4± 0.00a	35.0±0.02d	15.6± 0.78be
		N4	11.2± 0.01b	25.4±0.01f	13.0± 0.45ce
CT		N5	8.0± 0.05c	21.6±0.05h	18.2± 0.45bd
	LR	N0	2.4± 0.02h	24.2±0.14g	15.6± 0.00be
		N1	3.3± 0.02g	21.6±0.05h	15.6± 0.78bce
		N2	4.2± 0.02f	2.4±0.05p	15.6± 0.00be
		N3	1.1± 0.00k	2.7±0.03op	20.8± 0.90bc
		N4	1.0± 0.01k	4.8±0.00n	15.6± 0.00be
		N5	1.6± 0.03ij	3.9±0.01no	18.2± 0.45bd
	HR	N0	5.5± 0.01e	46.0±0.03a	18.2± 0.45bd
		N1	11.2± 0.01b	30.3±0.02e	30.0± 0.00ab
		N2	11.2± 0.01b	14.4±0.02j	20.8± 0.90bc
		N3	7.9± 0.01cd	13.2±0.03k	36.0± 0.00a
		N4	7.5± 0.06d	10.5±0.03l	30.0± 0.90ab
ST		N5	5.7± 0.03e	10.0±0.03l	28.0± 0.78bc
	LR	N0	3.3± 0.01g	17.1±0.13i	18.2± 0.45bd
		N1	3.2± 0.00g	10.8±0.03l	20.8± 0.45bc
		N2	1.4± 0.03ik	9.8±0.01l	23.4± 0.00b
		N3	1.7± 0.01ij	6.7±0.01m	20.8± 0.45bc
		N4	1.7± 0.01i	4.6±0.02n	10.4± 0.45de

	N5	1.2± 0.07jk	2.9±0.01op	7.8± 0.00e
LSD (P<0.05)		0.04	0.11	0.08

The interaction effects of tillage, residue, and N levels were significant for the $NaHCO_3$-Pi fraction (Table 1). The mean $NaHCO_3$-Pi fractions ranged from 2.4 to 46 mgkg-1 in treatment combinations (Table 2). Among all the tillage practices implemented, a significantly greater $NaHCO_3$-Pi fraction was observed in the CT (22.1 mgkg-1) plots compared to the ST plots (15.2 $mgkg^{-1}$) (Table 1). The application of CT-HR with lower N rates (N1: 60% RD and N2: 80% RD) considerably raised the soils' $NaHCO_3$-Pi fraction compared to CT-LR with similar N rates. The $NaHCO_3$-Pi decreased with the increase in N rates in combination with either ST-HR or ST-LR (Table 2). The treatment combination of ST-HR-N0 resulted in a significantly higher $NaHCO_3$-Pi fraction (46 $mgkg^{-1}$) than all other treatment combinations (Table 2). Likewise, the treatment interaction effect of the overall management practices for the $NaHCO_3$-Po fraction were significant (Table 1). The average value of $NaHCO_3$-Po fractions in soil varied from 7.8 to 36 $mgkg^{-1}$ (Table 2). Treatment combination of ST-HR-N3 demonstrated significantly higher $NaHCO_3$-Po fraction (36 $mgkg^{-1}$) while the N rate > N3 was observed with significantly lower $NaHCO_3$-Po fractions (Table 2).

Effects of management practices on moderately labile phosphorus fractions

Among all the tillage practices implemented, a significantly greater NaOH-Pi fraction was observed in the ST (24.1 $mgkg^{-1}$) plots compared to the CT plots (11.4 $mgkg^{-1}$) (Table 3). Likewise, the plots treated with HR were observed with significantly higher NaOH-Pi fraction (21.4 $mgkg^{-1}$) in comparison to LR treatment (Table 3). The NaOH-Pi fraction was significantly increased with N rates, being the highest in N5

(Table 4) combining with CT-HR, ST-HR and ST-LR treatments. The interactions effects of tillage, residue level and N rate significantly influenced NaOH-Pi fraction (Table 3). Higher residue coupled with higher N application had a significant positive effect on the NaOH-Pi in soils. Mean NaOH-Pi in soil samples ranged from 5.6 to 38.3 $mgkg^{-1}$, irrespective of treatments. The highest NaOH-Pi (38.3 $mgkg^{-1}$) was found in ST-HR-N5 treatment combination (Table 4).

Table 3. Effects of long-term combined application of different tillage, residue and N application treatments on moderately labile phosphorus fractions in wheat-mungbean-rice cropping system.

**Treatments**	**Moderately labile phosphorus fractions**	
**Tillage**	**NaOH-Pi**	**NaOH-Po**
CT	11.4±0.08b	18.5±0.17b
ST	24.1±0.17a	29.6±0.27a
Statistics	***	*
**Residue**		
HR	21.4±0.20a	31.9±0.26a
LR	14.0±0.10b	16.3±0.15b
Statistics	***	***
**Fertilizer**		
N0	10.3±0.21f	17.6±0.17b
N1	13.4±0.25e	16.3±0.13b
N2	16.1±0.31d	20.2±0.31b
N3	20.7±0.28c	28.6±0.50a
N4	22.3±0.26b	30.6±0.54a
N5	23.5±0.27a	31.2±0.53a
Statistics	***	***
Tillage x Residue	***	ns
Fertilizer x Tillage	***	**
Fertilizer x Residue	***	***
Fertilizer x Tillage x Residue	***	ns

CT= conventional tillage; ST= strip tillage; HR= higher residue: 30 cm of rice and wheat residue + 100% mungbean residue, by height; LR= lower residue: 15 cm of rice and

wheat residue + 100% mungbean residue; N0= 0% of recommended dose; N1= 60% of recommended dose; N2: 80% of recommended dose; N3: 100% of recommended dose; N4: 120% of recommended dose; N5: 140% of recommended dose

ns, *, **, *** indicate significant difference at the level P > 0.05, P < 0.05, P < 0.01, P < 0.001, respectively

Table 4. Moderately labile-P fractions in soil (depth: 0-15cm) under long term conventional (CT) and strip (ST) tillage systems; high residue (HR) and low residue (LR) retention; and six N application rates.

**Treatments**			**Moderately Labile Phosphorus Fractions**	
**Tillage**	**Residue**	**Fertilizer**	**NaOH-Pi (mgkg^{-1})**	**NaOH-Po (mgkg^{-1})**
CT	HR	N0	5.6±0.01r	20.8±0.45cg
		N1	8.6± 0.02p	18.2±0.45dh
		N2	9.7±0.01o	20.8±0.90cg
		N3	10.4±0.03o	28.6±0.90bd
		N4	13.4±0.00m	33.8±0.90b
		N5	16.6±0.03k	33.8±0.90b
	LR	N0	7.2±0.05q	15.6±0.78eh
		N1	5.7±0.01r	11.7±0.39fh
		N2	7.7±0.10pq	7.8±0.00h
		N3	17.8±0.05j	10.4±0.45gh
		N4	18.2±0.00j	10.4±0.45gh
		N5	15.2±0.02l	10.4±0.45gh
ST	HR	N0	22.3±0.03g	15.6±0.78eh
		N1	26.9±0.19e	18.2±0.45dh
		N2	33.2±0.09d	31.2±0.78bc
		N3	35.4±0.04c	52.0±0.45a
		N4	36.6±0.03b	54.6±0.78a
		N5	38.2±0.04a	54.6±0.78a
	LR	N0	6.1±0.05r	18.2±0.45dh
		N1	12.4±0.01n	17.1±0.25dh
		N2	13.8±0.01m	20.8±0.90cg
		N3	19.1±0.04i	23.4±1.35bf
		N4	21.1±0.01h	23.4±1.35bf
		N5	24.2±0.03f	26.0±1.19be
LSD (P<0.05)			0.09	1.28

The interaction effect for the NaOH-Po fractions among tillage, residues, and N levels was non-significant (Table 3), however, the individual treatment (tillage, residue retention, N rate) effect was significant. The soil NaOH-Po fractions ranged from 7.4 to 54.6 $mgkg^{-1}$ across all treatments (Table 4). The ST (29.6 $mgkg^{-1}$) plots showed a significantly greater NaOH-Po contents than the CT plots (18.5 $mgkg^{-1}$). Application of HR showed higher mean NaOH-Po fractions (31.9 $mgkg^{-1}$) than the LR (16.3 $mgkg^{-1}$). Mean NaOH-Po fractions increased with higher N rates in combinations with tillage and residue treatments. Among the N treatments, N5 recorded the highest mean value of NaOH-Po fractions which was statistically similar to N3 and N4 treatments. The study revealed a significant interaction between fertilizer and residue but non-significant interaction between tillage and residue for NaOH-Po fractions (Table 4).

Effects of management practices on non-labile phosphorus fractions

Acid-P contents in the soil samples varied from 2.0 mgkg-1 to 24.2 $mgkg^{-1}$ across all the treatments (Table 6). Strip tillage (ST) practice resulted in significantly higher acid-P fractions than CT treatments, however, residue treatments (HR, LR) had no significant influence on acid-P fractions. The interaction between tillage, residue level, and N fertilizers significantly influenced the acid-P fractions in soil (Table 5). Treatment combination of ST-HR-N1 produced the highest acid-P fractions, while the treatment combination ST-HR-N4 produced the lowest acid-P fractions.

Table 5. Effects of long-term combined application of different tillage, residue and N application treatments on non-labile phosphorus fractions in wheat-mungbean-rice cropping system.

**Treatments**	**Non-labile phosphorus fractions**	
**Tillage**	**Acid-P**	**Residue-P**
CT	12.3±0.09b	14.6±0.06a
ST	17.0±0.11a	17.1±0.18a
Statistics	*	ns
**Residue**		
HR	15.0±0.10a	13.5±0.10b
LR	14.3±0.12a	18.2±0.15a
Statistics	ns	*
**Fertilizer**		
N0	13.9±0.20b	20.6±0.25a
N1	17.2±0.16a	19.8±0.28ab
N2	14.9±0.14ab	16.5±0.24c
N3	17.2±0.06a	12.8±0.07d
N4	10.2±0.18c	17.5±0.14bc
N5	14.5±0.29b	7.8±0.11e
Statistics	***	***
Tillage x Residue	***	*
Fertilizer x Tillage	***	***
Fertilizer x Residue	ns	***
Fertilizer x Tillage x Residue	***	**

CT= conventional tillage; ST= strip tillage; HR= higher residue: 30 cm of rice and wheat residue + 100% mungbean residue, by height; LR= lower residue: 15 cm of rice and wheat residue + 100% mungbean residue; N0= 0% of recommended dose; N1= 60% of recommended dose; N2: 80% of recommended dose; N3: 100% of recommended dose; N4: 120% of recommended dose; N5: 140% of recommended dose

ns, *, **, *** indicate significant difference at the level P > 0.05, P < 0.05, P < 0.01, P < 0.001, respectively

Table 6. Non labile-P fractions in soil (depth: 0-15cm) under long term conventional (CT) and strip (ST) tillage systems; high residue (HR) and low residue (LR) retention; and six N application rates.

**Treatments**			**Non-labile Phosphorus Fractions**	
**Tillage**	**Residue**	**Fertilizer**	**Acid-P ($mgkg^{-1}$)**	**Residue-P ($mgkg^{-1}$)**
CT	HR	N0	7.0±0.09hi	13.3±0.14bf
		N1	10.7±0.12fh	15.8±0.29bd
		N2	10.0±0.00gh	12.5±0.43cf

		N3	14.5±0.09eg	15.6±0.000be
		N4	8.1±0.08hi	19.2±0.38b
		N5	10.0±0.00gh	9.7±0.06eg
	LR	N0	7.5±0.00hi	15.8±0.29bd
		N1	14.5±0.09eg	15.8±0.29bd
		N2	10.5±0.15fh	15.9±0.30bd
		N3	16.2±0.03be	11.6±0.06dg
		N4	15.0±0.15df	17.5±0.01bc
		N5	24.0±1.31a	12.4±0.03cf
ST	HR	N0	21.0±0.26ab	19.2±0.14b
		N1	24.2±0.14a	14.2±1.15bf
		N2	20.0±0.00ac	9.2±0.29fh
		N3	18.4±0.04be	11.7±0.38cg
		N4	15.5±0.39ce	15.8±1.04bd
		N5	20.5±0.09ab	5.8±0.14gh
	LR	N0	20.0±0.02ac	34.2±0.14a
		N1	19.4±0.05ad	33.3±0.38a
		N2	19.1±0.04be	28.3±0.38a
		N3	19.9±0.03ac	12.5±0.22cf
		N4	2.0±0.03j	17.5±0.01bc
		N5	3.7±0.05ij	3.3±0.03h
LSD (P<0.05)			0.48	0.6

The average residue-P fractions ranged from 3.3 mgkg^{-1} to 34.2 mgkg^{-1} across all the treatment combinations. The individual tillage treatment has no significant impact on the residue-P fraction, however, the interaction of tillage, residue levels and N fertilizers rates significantly influenced residue-P fraction (Table 5). Lower residue incorporation increased residue-P fraction (18.2 mgkg^{-1}) in soil than higher residue (13.5 mgkg^{-1}) treated plots. When compared with other treatment combinations, ST-LR in combination with lower N rate (<N3) raised residual-P fractions in soils. Among all the treatment combinations, ST-LR-N0 had the maximum residue-P concentration and the minimum was found in ST-LR-N5 (Table 6).

Discussions

After 36 crop cycles, soluble P levels varied with N treatment rates, with the optimal rate yielding the highest soluble P content. The most bioavailable P pool in soil is the labile P fractions, which is made up of soluble P, $NaHCO_3$-Po, and $NaHCO_3$-Pi (31. Blake et al., 2003). The labile P fractions were higher in 100% RD or lower rates, irrespective of tillage and residue retention levels. By contrast the higher N rate than 100% resulted in lower labile P pools in the current study. Reduced fertilizer-soil mixing under ST would have reduced P retention, allowing more P to remain in soluble form in the soil solution. According to 32. Hu et al. (2010), nitrogen addition promotes potential net N mineralization and potential net nitrification processes which may raise the biotic P requirement. It is likely that increased N rate may have resulted in an increased uptake of available P as higher N rate than 100% increased crop yields and nutrient uptake (33. Kader et al., 2022). The drop in soil $NaHCO_3$-Pi concentration may be attributed to the N addition, which promoted plant development and root P uptake and was subsequently distributed to other plant parts (34. Zhao et al., 2010). After N is added, the proportion of labile P may decrease because microorganisms and plants typically use labile P first to meet their increased P demands. Labile P fractions, due to its more mobility, may have been leached out with the percolating water. Thus, application of optimum N with CA practices can reduce the possibility of eutrophication, groundwater toxicity and nitrogenous greenhouse gas emissions. 35. Fan et al. (2018) reported that soluble P is linked to soil organic compounds and organic acids and is poorly adsorbed to Fe and Al at mineral surfaces. In the current experiment, higher N rate decreased labile P fractions which also might be due to soil acidity caused by higher urea application rate. Ammonium based nitrogenous fertilizers can lower soil pH and decrease phosphorus availability because of fixation. Moreover, ammonium ions (NH_4^+) from urea fertilizers

can interact with soil particles, leading to increased adsorption of labile-P fractions that are accessible. Other past research has also argued that the soluble-P may be decreased as a result of P fixation, leaching and low phosphate ion mobility (5. Amaizah et al., 2012; 36. Bolo et al., 2021), because higher soluble-P is the result of a synergistic impact between increased accessible Pi and increased N (37. Anil et al., 2022). Soluble P increased with high residue retention, likely because additional residue reduced P adsorption on specific minerals, enhancing soluble P availability (37. Anil et al., 2022; 38. Haokip et al., 2020; 39. Nunes et al., 2020; 40. Sharma et al., 2020). Our results are also in line with 40. Sharma et al. (2020) who indicated that tillage treatments did not significantly alter the soluble P content, but the application of residues and N levels did. According to 41. Hou et al. (2018), soluble-P may rise as a result of soil organic matter buildup as dissolved soil organic matter can restrict P sorption and enhance P solubility in soil. Past research has also supported this argument where lower N rate corresponded with higher soil organic carbon accumulation (42. Jahangir et al., 2021).

The physiologically accessible inorganic P fraction is $NaHCO_3$-Pi, while $NaHCO_3$-Po is thought to be the readily mineralized P fraction in the form of nucleic acids and phospholipids (43. Sharma et al., 2022). The proportions of $NaHCO_3$-Pi were greatly impacted by tillage and residue treatments and their interactions with N treatments where ST with HR coupled with lower N rate increased the $NaHCO_3$-Pi suggesting that CA is favorable for more available P fractions. Mean $NaHCO_3$-Pi content was found considerably higher with total CA implementation (reduced tillage, higher residue retention) than with partial CA implementation (reduced tillage, lower residue retention) (38. Haokip et al., 2020). It is likely that along with $NaHCO_3$-Pi, CA also increase in the organic P contents (44. Sharpley et al., 2004; 45. Hao et al., 2008).

However, our data suggests that the significant effect of CA on labile P fractions are mainly driven by the crop residues rather than the tillage practices. Only a small portion of applied P fertilizers can be taken up by plants, while the majority is fixed in soil in different less-available forms (46. Dwivedi et al., 2017) indicating that application of excessive P will likely to enhance P loss via leaching as continuous application of excessive P may cause saturation of fixation sites. 4. Meena et al. (2013) also reported a noteworthy alteration in the $NaHCO_3$-Pi and NaOH-Pi percentages due to tillage and residue management. Previous researches observed that reduced soil disturbance (such as zero tillage, minimum tillage) and crop residue retention on the soil surface resulted in higher labile P accumulation in the surface layer (14. Reddy et al., 2014; 15. Kumawat et al., 2018; 47. Redel et al., 2007). Thus, addition of chemical P fertilizer could be lowered under CA practices to avoid P loss from soils through leaching or fixation.

Increased N rate with conservation agriculture (ST-HR combination) increased the moderate P pools in soils. Moderately labile P (NaOH-Pi and NaOH-Po) are potentially available P fractions that are loosely held with Fe and Al at clay surfaces by chemisorption or linked to humic substances (48. Cross and Schlesinger, 1995). The NaOH-Pi fraction is generally considered the less readily available P and may represent P adsorption sites. The equilibrium between labile and moderately labile P is faster than the equilibrium between labile and non-labile P (4. Meena et al., 2013). The current investigation found that CA with increased N rate had a higher NaOH-Pi concentration, which was consistent with previous research findings (49. Mahmood et al., 2021; 50. Yang et al., 2015; 51. Qaswar et al., 2021; 52. Chen et al., 2018). When N fertilization increased soil NO_3^- –N contents, it could progressively convert residual P and labile P fractions into NaOH-Pi, that's why there was an increase in NaOH-Pi in response to N

fertilization. The reasons might be soil pH changes, microbial dynamics, ionic interactions, and redox conditions that are influenced by increased NO_3^-–N concentrations. Increased soil NO_3^-–N released from ammonium-based fertilizers might acidify the soil, influencing the solubility of P. Higher NO_3^- ions could enhance microbial activity that convert organic phosphorus to inorganic forms. Additionally, presence of higher NO_3^-–N might alter the adsorption-desorption equilibrium, potentially converting soil phosphorus from residual P or labile P forms into NaOH-Pi. 37. Anil et al. (2022) also reported greater moderately labile and non-labile organic P under CA practices. Opposite to our results, 53. Sun et al. (2022) and 54. Jing et al. (2021) demonstrated different results of considerable drop in the NaOH-Pi pool sizes in the N application treatments as compared to the control treatment. Higher NaOH-Pi pools with higher N rates in our study can be attributed to mobilization of NaOH-Pi fraction to augment the sufficient amount of accessible Pi for crop growth. The decline in NaOH-Pi pools indicated that the size of the $NaHCO_3$-Pi pool in the soil might not be sufficient to meet crop P needs (55. Hassan et al., 2012) and thus converted to labile inorganic P fractions. Also, through P mineralization, the NaOH-Po pool may serve as the primary source of plant-available P in the soil and sustain plant-available P levels (56. Beck and Sanchez, 1994). Lower mineralization would be expected at lower N supply and as a result, lower N rate resulted in lower NaOH-Po pools. In situations where soil P supply is low, Po mineralization serves as crops' primary source of available inorganic P resulting in lower NaOH-Po pools (57. Liao et al., 2020). Compared to conventional agriculture, CA improved the NaOH-Pi and NaOH-Po fractions (38. Haokip et al., 2020) which was also evident in the present study. Crops used the inorganic P produced through hydrolyzing the NaOH-Po pool, or it was converted into

other Pi pools (NaOH-Pi or $NaHCO_3$-Pi). Moreover, it is likely that surface-retained leftovers may have biologically immobilized soil Pi, leading to a rise in NaOH-Po in HR treatment. Increased microbial activity might be aided by the addition of residues, resulting in accumulation of NaOH-P (both Pi and Po) by mineralization, breakdown of Fe and or Al chelation with P. Remainder of crop residues in residue retention and incorporation treatments likely converted part of the soil Pi extracted by crops to Po (14. Reddy et al., 2014). Proper management practices should be undertaken for preventing loss of moderately labile-P pools through erosion, runoff or other factors.

The levels of acid-P varied depending on the tillage and residue management coupled with N rate. Recalcitrant-P fractions, known as acid-P and residual-P, are thought to be poorly labile and difficult for plants to obtain. The acid-P fraction contained P bonded to Ca (48. Cross and Schlesinger, 1995) and the quantity is high in alkaline soil due to the high stability of Ca-P (37. Anil et al., 2022). Opposite to our findings, lower acid-P was discovered under CA (minimal tillage, residue retention) by 38. Haokip et al. (2020). They also found that there was no significant interaction between tillage and nutrients on acid-P since this fraction was stable and insoluble. In the experimental site, soil pH was close to neutral and the acid-P pools under different management didn’t follow any trends. The acid-P fractions' altered concentrations suggest that these fractions may be involved in long-term P cycling. Under CA, the acid-P fractions may function as a slow-release storage of phosphorus, ensuring that plants constantly have a supply of available P even when short-term inputs are altered, supporting long-term soil fertility and crop production over multiple growing seasons. In N fertilization, acid-P concentrations were higher than in control, with the exception of N4 and N5 treatments. The acid P pool was reduced in the N rate > N3 compared to

the other treatments might suggest that the pool was hydrolyzed as hydrolysis of a relatively resistant acid-P pool could be aided by the excessive rate of N application (54. Jing et al., 2021). Since crop demand for P absorption could be increased by high N fertilizer, it is possible that acid-P depletion occurred in addition to labile Pi fractions (50. Sun et al., 2022). A portion of the acid-P pool's hydrolysis products would move straight into the $NaHCO_3$-Pi pool in this study. Nevertheless, the portions of Pi near the surface of the original mineral particles might easily be re-adsorbed and fixed again and merged acid-P if the hydrolyzed free P or the immediately generated $NaHCO_3$-Pi cannot be absorbed by crops or microorganisms in time; and it is also possible for other P pools to partially complement acid-P pools (54. Jing et al., 2021). Through proper cultural management, these non-labile P pools can be utilized over the multiple cropping seasons and chemical P inputs can be minimized.

The native mineral P and extremely refractory forms of P that make up the residual-P pool are unlikely to provide short-term contributions to the soil solution and plant nutrients (58. Shi et al., 2013). Our results showed that the residual-P declined in the high N treatment compared to the control treatment, which was in line with other findings (49. Mahmood et al., 2021; 35. Fan et al., 2018). The reason behind this might be enhanced soil microbial activity, that was stimulated with N addition and the release of phosphorus from residual P pools by those microbes. Lower residues might have boosted residual-P at lower N rates, whereas higher residues might have decreased its mobilization due to the residual-P's persistence, resistance, and insoluble nature. The gradual decrease in residual P suggests that, in the event of prolonged and ongoing cultivation, residual-P in soils could change into more labile forms (58. Shi et al., 2013) which may be true in this study as both of them were found higher in the same treatment

combinations (ST-LR with < N3 of N rate). When agricultural residues are added to soil systems, the residual P portion could function as a sink (45. Hao et al., 2008). The drop in residual-P during higher N rates might also have resulted from an increase in biomass among the plants (particularly root length and root hair density) during P shortage which enhanced plant performance and greatly increased residual P absorption (59. Irfan et al., 2020; 60. McDowell et al., 2016).

Conclusions

A larger amount of resistant phosphorus (non-available) storage under conventional and conservation agriculture (CA) practices was obtained with the moderate nitrogen (N) treatment. However, the significantly higher rates of N reduced this amount of resistant or stored P possibly by greater mineralization, plant uptake and moving down to the deeper soil layers. Long-term practices of CA with higher N rate increased the moderately labile P fractions suggesting that CA help in the buildup of soil-P fractions and this could eventually reduce P fertilizer application. Excessive rate of N application enhanced transformation of stable P to moderately labile P (inorganic), possibly by changing the soil conditions and microbial community. In addition to that, CA increased the total P fractions mainly by addition of organic P through crop residues. The observed data from this long-term CA experiment suggests that the significant effect of CA on labile P fractions is mainly driven by the crop residues rather than the tillage practices. But this additional crop residue can enhance the environment related problems like groundwater toxicity, eutrophication, volatilization loss and nitrous oxide emissions etc. upon mineralization, which should be kept in mind during agricultural management. Further researches are necessary for enhancing availability of both the soil nutrients (N

and P) along with mitigating environmental pollution. Moderately labile organic P can be transformed to the labile inorganic P in the lower N rates. These findings may contribute to better understand dynamics and fractions of P in soil, which could help for optimizing N and P management strategies in the farmers' fields both in Bangladesh agroecological zone-9 conditions and in other regions of the world with similar environmental conditions. Long term conservation agriculture and optimum nitrogen application could be a sustainable way to supply adequate amount of available phosphorus contents for plants uptake over the years and to maintain soil resilience and crop productivity. Furthermore, these practices could be integrated in future with biochar application or microbial inoculants according to specific soil conditions for getting better outcome from limited natural resources.

Acknowledgment

The authors gratefully acknowledge the logistic support from both the Department of Soil Science at Bangladesh Agricultural University, and Khulna Agricultural University to carry out this research work.

References

1. Hu B, Yang B, Pang X, Bao W, Tian G. Responses of soil phosphorus fractions to gap size in a reforested spruce forest. Geoderma. 2016; 279: 61–69.

2. Liu Y, Feng L, Hu H, Jiang G, Cai Z, Deng Y. Phosphorus release from low grade Rock phosphates by low molecular weight organic acids. J Food Agric Environ 2012; 10: 1001-1007.

3. Williams A, Bo¨rjesson G, Hedlund K. The effects of 55 years of different inorganic fertiliser regimes on soil properties and microbial community composition. Soil Biol Biochem. 2013; 67:41–6.

4. Meena AL, Pandey RN, Behera UK, Meena MD. Effect of tillage and crop residue management on phosphorus transformation and phosphatase enzyme activity under pigeonpea (*Cajanus cajan*) rhizosphere and non-rhizosphere soil. Annals Biol. 2013; 29 (2): 148–57.

5. Amaizah NR, Čakmak D, Saljnikov E, Roglić G, Mrvic V, Krgović R, Manojlović DD. Fractionation of soil phosphorus in a long-term phosphate fertilization. J Serb Chem Soc. 2012; 77(7): 971-981.

6. Tang X, Li Z, Ma Y, Liang Y. Mechanism of fulvic acid-and organic manure-mediated phosphorus mobilization in black soil at low temperature. J Plant Nutri Fertilizers. 2012; 18: 893–899.

7. Andersson JA, Giller KE. On heretics and God's blanket salesmen: contested claims for conservation agriculture and the politics of its promotion in African smallholder farming. In: Sumberg J, Thompson J, editors. Contested agronomy: agricultural research in a changing world. Taylor & Francis; 2012.

8. Powlson DS, Stirling CM, Thierfelder C, White RP, Jat ML. Does conservation agriculture deliver climate change mitigation through soil carbon sequestration in tropical agro-ecosystems? Agric Ecosys Environ. 2016; 220: 164– 174.

9. Kassam AH, Friedrich T, Shaxson F, Pretty J. The spread of Conservation Agriculture: justification, sustainability and uptake. Int J Agric Sustainability. 2009; 7: 1-29.

10. Tiefenbacher A, Sandén T, Haslmayr HP, Miloczki J, Wenzel W, Spiegel H. Optimizing carbon sequestration in croplands: A synthesis. Agron J. 2021; 11: 882.

11. Brouder SM, Gomez-Macpherson H. The impact of conservation agriculture on smallholder agricultural yields: a scoping review of the evidence. Agric Ecosys Environ. 2014; 187: 1-32.

12. Palm C, Blanco-Canqui H, DeClerck F, Gatere L, Grace P. Conservation agriculture and ecosystem services: an overview. Agric Ecosys Environ. 2014; 187: 87–105.

13. Singh NJ, Athokpam HS, Patel KP, Meena MC. Effect of nitrogen and phosphorus in conjunction with organic and micronutrients on yield and nutrient uptake by maize-wheat cropping sequences and soil fertility. Environ Ecol. 2009; 27: 25-31.

14. Reddy D, Kushwah S, Srivastava S, Khamparia RS. Long-term wheat residue management and supplementary nutrient input effects on phosphorus fractions and adsorption behavior in a Vertisol. Communications Soil Sci Plant Analysis. 2014; 45: 541-554.

15. Kumawat C, Sharma VK, Meena MC, Dwivedi BS, Barman M, Kumar S, Chobhe KA, Dey A. Effect of crop residue retention and phosphorus fertilization on P use efficiency of maize and biological properties of soil under maize-wheat cropping system in an Inceptisol. Indian J Agric Sci. 2018; 88: 1184-1189.

16. Kumar A, Behera UK, Dhar S, Shukla L, Bhatiya A, Meena MC et al. Effect of tillage, crop residue and phosphorus management practices on the productivity and profitability of maize cultivation in Inceptisols. Indian J Agric Sci. 2018; 88: 1558–1167. https://doi.10.56093/ijas.v88i10.84223

17. Haokip IC, Dwivedi BS, Meena MC, Datta SP, Sharma VK, Saharawat YS. Effect of phosphorus fertilization and microbial inoculants on yield, phosphorus use-efficiency and available phosphorus in maize – wheat cropping system. Ind J Agric Sci. 2019; 89: 806-12.

18. Bunemann EK, Heenan DP, Marschner P, McNeill AM. Long-term effects of crop rotation, stubble management and tillage on soil phosphorus dynamics. Australian J Soil Res. 2006; 44: 611–18.

19. Zhu X, Chen H, Zhang W, Huang J, Fu S, Liu Z, Mo J. Effects of nitrogen addition on litter decomposition and nutrient release in two tropical plantations with N_2-fixing vs. non-N_2-fixing tree species. Plant Soil. 2016; 399(1): 1–14.

20. Mayakaduwage S, Alamgir M, Mosley L, Marschner P. Phosphorus pools in sulfuric acid sulfate soils: influence of water content, pH increase and P addition. J Soils Sediments. 2020; 20: 1446–1453. https://doi.org/10.1007/s11368-019-02521-1

21. Carreira JA, García-Ruiz R, Liétor J, Harrison AF. Changes in soil phosphatase activity and P transformation rates induced by application of N- and S-containing acid-mist to a forest canopy. Soil Biol Biochem. 2000; 32(13): 1857–1865.

22. Duncan EG, O'Sullivan CA, Roper MM, Biggs JS, Peoples MB. Influence of co-application of nitrogen with phosphorus, potassium and sulphur on the apparent efficiency of nitrogen fertiliser use, grain yield and protein content of wheat: review. Field Crops Res. 2018; 226: 56–65.

23. Dhillon J, Torres G, Driver E, Figueiredo B, Raun WR. World phosphorus use efficiency in cereal crops. Agronomy J. 2017; 109: 1670–1677.

24. Egashira K, Takenaka J, Shuto S, Moslehuddin AZM. Phosphorus status of some paddy soils in Bangladesh. Soil Sci Plant Nutr. 2003; 49(5): 751-757.

25. Chang SC, Jackson ML. Fractionation of soil phosphorus. Soil Sci. 1957; 84:133–144.

26. Hingston FJ, Atkinson RJ, Posner AM, Quirk JP. Specific adsorption of anions. Nature. 1967; 215(5109): 1459–1461.

27. Popović AR, Đorđević D. Speciation of selected trace and major elements in lignite used in'Nikola Tesla A'power plant (Obrenovac, Serbia). J Serb Chem Soc. 2005; 70(12): 1497-1513.

28. Hedley MJ, Stewart JWB, Chauhan BS. Changes in inorganic and organic soil phosphorus fractions induced by cultivation practices and by laboratory incubations. Soil Sci Soc Am J. 1982; 46: 970–976.

29. Tiessen H, Moir JO. Characterization of available P by sequential extraction. In: Carter MR, Gregorich EG. Editors. Soil sampling and methods of analysis,

chapter 25, 2nd ed. Canadian Society of Soil Science. Boca Raton Fl, USA; 2008. pp. 293–306.

30. FRG, Fertilizer Recommendation Guide. Bangladesh Agricultural Research Council. Khamarbari road, Farmgate, Dhaka-1215. 2018.

30. Saleque MA, Naher UA, Islam A, Pathan ABMBU, Hossain ATMS, Meisner CA. Inorganic and organic phosphorus fertilizer effects on the phosphorus fractionation in wetland rice soils. Soil Sci Soc Am J. 2004; 68(5): 1635–1644.

31. Blake L, Johnston AE, Poulton PR, Goulding KWT. Changes in soil phosphorus fractions following positive and negative phosphorus balances for long periods. Plant Soil. 2003; 254: 245–261.

32. Hu YL, Zeng DH, Liu YX, Zhang YL, Chen ZH, Wang ZQ. Responses of soil chemical and biological properties to nitrogen addition in a Dahurian larch plantation in Northeast China. Plant Soil. 2010; 333: 81–92.

33. Kader MA, Jahangir MMR, Islam M, Begum R et al. Long-term conservation agriculture increases nitrogen use efficiency by crops, land equivalent ratio and soil carbon stock in a subtropical rice-based cropping system. Field Crops Res. 2022; 287:108636.

34. Zhao Q, Liu XY, Hu YL, Zeng DH. Effects of nitrogen addition on nutrient allocation and nutrient resorption efficiency in *Larix gmelinii*. Scientia Silvae Sinica. 2010; 46:14–19.

35. Fan Y, Lin F, Yang L, Zhong X, Wang M, Zhou J, Chen Y, Yang Y. Decreased soil organic P fraction associated with ectomycorrhizal fungal activity to meet increased P demand under N application in a subtropical forest ecosystem. Biol Ferti Soils. 2018; 54(1): 149–161. https://doi.org/10.1007/s00374-017-1251-8

36. Bolo P, Kihara J, Mucheru-Muna M, Njeru EM, Kinyua M, Sommer R. Application of residue, inorganic fertilizer and lime affect phosphorus solubilizing microorganisms and microbial biomass under different tillage and cropping systems in a Ferralsol. Geoderma. 2021; 390: 114962.

37. Anil AS, Sharma VK, Jiménez-Ballesta R, Parihar CM, Datta SP, Barman M, Chobhe KA, Kumawat C, Patra A, Jatav SS. Impact of long-term conservation agriculture practices on phosphorus dynamics under maize-based cropping systems in a sub-tropical soil. Land. 2022; 11: 1488.

38. Haokip IC, Dwivedi BS, Meena MC, Datta SP, Jat HS, Dey A, Tigga P. Effect of conservation agriculture and nutrient management options on soil phosphorus fractions under maize-wheat cropping system. J Indian Soc Soil Sci. 2020; 68(1): 45–53. https://doi.org/10.5958/0974-0228.2020.00005.5

39. Nunes R, de S, de Sousa DMG, Goedert WJ, de Oliveira LEZ, Pavinato PS, Pinheiro TD. Distribution of soil phosphorus fractions as a function of long-term soil tillage and phosphate fertilization management. Frontiers Earth Sci. 2020; 8. https://doi.org/10.3389/feart.2020.00350

40. Sharma KL, Indoria AK, Srinivas K, Reddy KS, Charry GR, Lal M, Srinivasarao C, Chandrika DS, Osman M, Prasad JVNS, Balloli SS, Pratibha G, Parmar B, Veni

VG, Pushpanjali P, Vasavi M, Haindavi P, Gayatri DLA. Effect of long-term use of tillage, crop residue and N application on phosphorus fractions in soil under sorghum (*Sorghum vulgare* L.)-castor System (*Ricinus communis*) in Rainfed Alfisol Soils. Commun Soil Sci Plant Analys. 2020; 51(1): 1–13.

41. Hou E, Tang S, Chen C, Kuang Y, Lu X, Heenan M, Wen D. Solubility of phosphorus in subtropical forest soils as influenced by low-molecular organic acids and key soil properties. Geoderma. 2018; 313:172-180.

42. Jahangir MMR, Nitu TT, Uddin S, Siddaka A, Sarker P, Khan S et al. Carbon and nitrogen accumulation in soils under conservation agriculture practices decreases with nitrogen application rates. Applied Soil Ecol. 2021; 168: 104178. https://doi.org/10.1016/j.apsoil.2021.104178

43. Sharma S, Kaur S, Parkash Choudhary O, Singh M, Al-Huqail AA, Ali HM, Kumar R, Siddiqui MH. Tillage, green manure and residue retention improves aggregate-associated phosphorus fractions under rice–wheat cropping. Scientific Reports. 2022; 12(1). Available: https://doi.org/10.1038/s41598-022-11106-x

44. Sharpley AN, McDowell RW, Kleinman PJ. Amounts, forms, and solubility of phosphorus in soils receiving manure. Soil Sci Soc Am J. 2004; 68: 2048–2057.

45. Hao X, Godlinski F, Chang C. Distribution of phosphorus forms in soil following long term continuous and discontinuous cattle manure applications. Soil Sci Soc Am J. 2008; 72: 90–97.

46. Dwivedi BS, Singh VK, Shekhawat K, Meena MC, Dey A. Enhancing use efficiency of phosphorus and potassium under different cropping systems of India. Indian J Fertilisers. 2017; 13: 20–41.

47. Redel YD, Rubio R, Rouanet JL, Borie F. Phosphorus bioavailability affected by tillage and crop rotation on a Chilean volcanic derived Ultisol. Geoderma. 2007; 139: 388-396.

48. Cross AF, Schlesinger WH. A literature review and evaluation of the Hedley fractionation: applications to the biogeochemical cycle of soil phosphorus in natural ecosystems. Geoderma. 1995; 64: 197- 214.

49. Mahmood M, Tian Y, Ma Q, Hui X, Elrys AS, Ahmed W, Mehmood S, Wang Z. Changes in phosphorus fractions in response to long-term nitrogen fertilization in loess plateau of China. Field Crops Res. 2021; 270.

50. Yang, K, Zhu J, Gu J, Yu L, Wang Z. Changes in soil phosphorus fractions after 9 years of continuous nitrogen addition in a Larix gmelinii plantation. Annals Forest Sci. 2015; 72(4): 435–442. https://doi.org/10.1007/s13595-014-0444-7

51. Qaswar M, Jing H, Ahmed W, Abbas M, Dongchu L. Linkages between ecoenzymatic stoichiometry and microbial community structure under long-term fertilization in paddy soil: a case study in China Linkages between ecoenzymatic stoichiometry and microbial community structure under long-term fertilization. Applied Soil Ecol. 2021; 161: 103860. https://doi.org/10.1016/j.apsoil.2020.103860

52. Chen H, Chen M, Li D, Mao Q, Zhang W, Mo J. Responses of soil phosphorus availability to nitrogen addition in a legume and a non-legume plantation. Geoderma. 2018; 322: 12–18.

53. Sun W, Villamil MB, Behnke GD, Margenot AJ. Long-term effects of crop rotation and nitrogen fertilization on phosphorus cycling and balances in loess-derived Mollisols. Geoderma. 2022; 420.

54. Jing D, Yan Y, Ren T, Lu J, Wang X, Tan W, Liu F, Jaisi DP, Feng X. Effects of nitrogen application rate on phosphorus transformation in an Alfisol: Results from phosphate-oxygen isotope ratios. Applied Geochem. 2021; 134: 105094.

55. Hassan HM, Marschner, P, McNeill A, Tang C. Grain legume pre-crops and their residues affect the growth, P uptake and content of P pools in the rhizosphere of the following wheat. Biol Fert Soils. 2012; 48: 775-785.

56. Beck MA, Sanchez PA. Soil phosphorus fraction dynamics during 18 years of cultivation on a Typic Paleudult. Soil Sci Soc Am J. 1994; 58: 1424–1431.

57. Liao D, Zhang C, Li H, Lambers H, Zhang F. Changes in soil phosphorus fractions following sole cropped and intercropped maize and faba bean grown on calcareous soil. Plant Soil. 2020; 448: 587–601. https://doi.org/10.1007/s11104-020- 04460-0

58. Shi Y, Ziadi N, Messiga AJ, Lalande R, Hu Z. Changes in soil phosphorus fractions for a long-term corn-soybean rotation with tillage and phosphorus fertilization. Soil Sci Soc Am J. 2013; 77(4): 1402–1412.

59. Irfan M, Aziz T, Maqsood MA, Bilal HM, Siddique KHM, Xu M. Phosphorus use efficiency in rice is linked to tissue-specific biomass and P allocation patterns. Scientific Reports. 2020; 10: 1–15. Available: https://doi.org/10.1038/s41598-020-61147-3

60. McDowell RW, Condron LM, Stewart I. Variation in environmentally- and agronomically-significant soil phosphorus concentrations with time since stopping the application of phosphorus fertilisers. Geoderma. 2016; 280: 67–72.

Click here to access/download;Figure;Figure1.tif

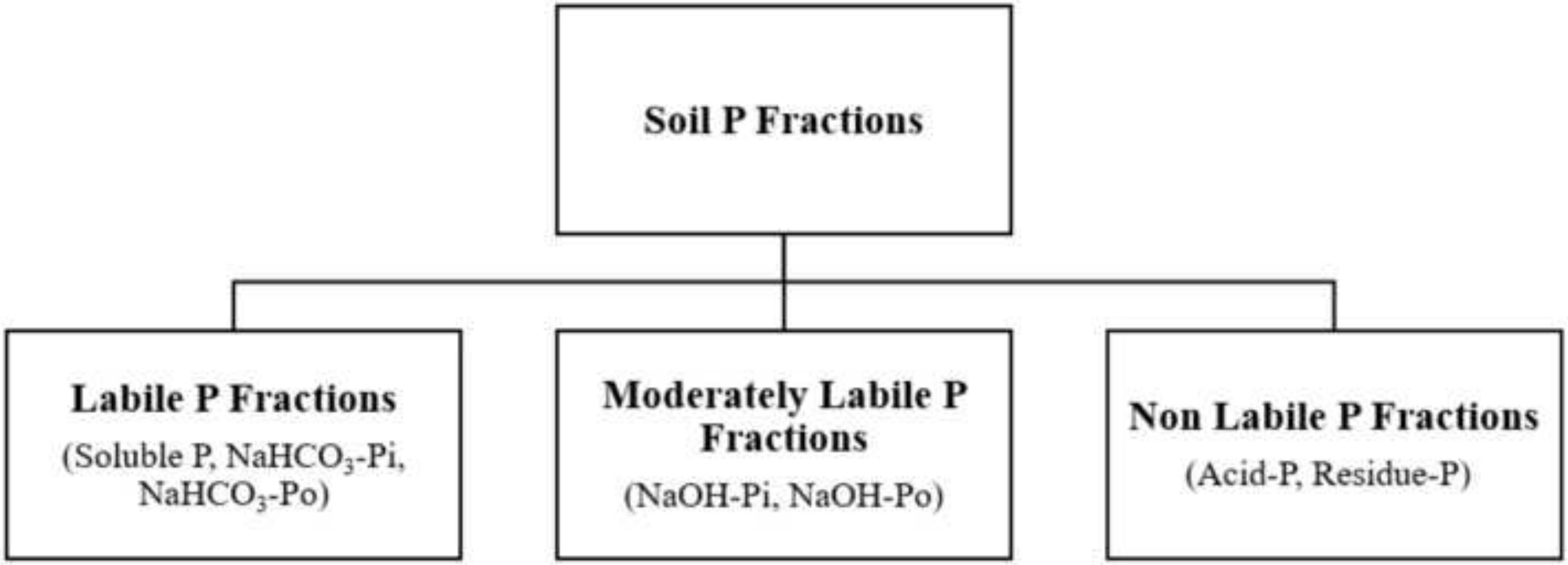

Supplement: Attachment — Submitted filename: PONE-D-25-17789.pdf [file pone.0333013.s003.pdf]
