# Supplementary material for: Long-term conservation agriculture with optimum nitrogen fertilization improves soil phosphorus availability
Source: PLoS One. 2025 Sep 24;20(9):e0333013. doi: 10.1371/journal.pone.0333013 (PMC12459793; doi:10.1371/journal.pone.0333013)
Supplement: Attachment — Submitted filename: Response to Reviewers.pdf [file pone.0333013.s005.pdf]

Response to Reviewers Comments Manuscript # PONE-D-25-17789

Dear Dr. Abhay Omprakash Shirale,

Thank you for giving us the opportunity to submit the revised manuscript entitled "Effect of long-term conservation agriculture with nitrogen rates on soil available and labile phosphorus pools" for publication in *PLOS ONE*. We appreciate the considerable time and efforts you and the reviewers put into providing suggestions on our manuscript. We incorporated the reviewers' suggestions in our manuscript.

Reviewer 1

1. Comment: Hypothesis of the work is not well formulated in the 'Introduction' section. The authors did not present a novel justification for carrying out this study. What is the hypothesis of the present study?

   Author response: Thank you for pointing this out. we have added the hypothesis of the present study in the text and also a clear justification for this hypothesis.

2. Comment: Introduction is insufficient and needs more improvement. The novelty of the work must be identified and stated more carefully. The authors have to try to explain why this paper is relevant to the wider readership. Authors should show the limitations of previous papers.

   Author response: CA system has been recognized to change the nutrient dynamics and stoichiometry but how these dynamics interact with N rate for P availability in floodplain soils in subtropical agroecosystems are limited. We added the limitations of previous research works and also added the novelty of our research in the text.

3. Comment: Result should be written in concise way.

   Author response: We appreciate the reviewer's feedback. The result section has been rewritten following the valuable comments of the reviewers.

4. Comment: Quality of figures should be improved.

   Author response: The quality of figure has been improved and a new one is added.

5. Comment: Author should try to strengthen the discussion part. Put reason why this type of result is obtained.

   Author response: We have rewritten this section and improved the quality of the section with science-based arguments on the findings.

6. Comment: Authors need to rephrase the "Conclusions" section. Add some limitations, underscore the scientific value added of your paper, and/or the applicability of your findings/results and future scope of study.

   Author response: We have added the suggested contents on conclusion section of the manuscript.

7. Comment: Overall, the manuscript needs extensive language editing and formatting. There are so many typography mistakes in the manuscript.

   Author response: Thank you for this suggestion. The manuscript has been thoroughly edited by a native English speaker, Dr. Richard Bell, Professor at Murdoch University, Australia. The readability and English grammar and use of the article are now improved.

Reviewer 2

1. Comment: In title what type of “effect” should be specified?

   Author response: Thank you for pointing this out. We have made a correction in the title.

2. Comment: Title words should not be keywords.

   Author response: We have added new keywords different from title words.

3. Comment: Hypothesis is not clear and results should be measurable (% increase or decrease) in abstract. Higher N rates did not reduce all P fractions.

   Author response: Abstract section has been updated following the comments of reviewers.

4. Comment: There are too many irrelevant topics included in introduction section. There are huge references on. But your references are too old. Lack of novelty and research scope. Introduction should be rearranged by the sequence of - important of the research, what is the known about the topic, unknown about the topic, why this research is important and finally aims of this research.

   Author response: We rewritten the section by rearranging the sequence, adding some new information following the reviewer’s suggestions.

5. Comment: Line 112: AEZ 9 is Old Brahmaputra Floodplain not Old Himalayan Piedmont Plain. Line 144: Medium Highland Sonatala soil series is poorly drained soil not moderately drained.

   Author response: Thank you for pointing these out. We have now made the necessary corrections in the manuscript.

6. Comment: Line 151-152: What are the crops diversifications? Provide the 12 years cropping pattern.

   Author response: Thank you for your feedback. Although we agree that this is an important consideration, we didn't provide 12yrs cropping pattern in details in this manuscript because it was already mentioned that a wheat–mungbean–rice cropping pattern was continued at our experimental location for 12 years with the same management to understand the long-term effects of CA on soil biogeochemistry and crop productivity.

7. Comment from Reviewer 2: Line 162: RD? Elaborate first.

   Author response: We elaborated the term in the manuscript.

8. Comment: 168-171: Fertilizers doses for each crop are not clear. Provide in a table and give it to supplementary data.

   Author response: While we appreciate the reviewer's feedback, but the given reference has explained the crop management in a great detail.

9. Comment: 180-182: 0-15 soil depth? Sample collection procedure should be clear specially core sampler.

   Author response: We revised the methodology and mentioned that the samples were collected with the help of an auger at 0-15 cm soil depth.

10. Comment: Need to rewrite the results section.

    Author response: The result section has been rewritten following the valuable comments of the reviewers.

11. Comment: Discussion is not another result. The discussion should link all of your findings. Each paragraph should be well structured and interconnected.

    Author response: We have rewritten this section and tried to improve the quality and strengthen the arguments for the obtained results.

12. Comment: conclusions must be self-explanatory. This section should highlight the novelty and implication of your study also.

    Author response: The conclusion section has been rewritten following the valuable comments of the reviewers.

13. Comment: Avoid unnecessary tables and figures and they must be self-explanatory.

Author response: We think the figures makes a valuable contribution to the methodology section. But we have removed some of the tables that are unnecessary and made some corrections to improve the readability and visibility of data.

14. Comment: English improvement is needed in whole MS.

Author response: The manuscript has been edited by a native English speaker, Dr. Richard Bell, Professor, Murdoch University, Australia.

Reviewer 3

1. Comment: The abstract should be focused and well-structured, offering the reader a clear snapshot of the study's purpose and outcomes.

Author response: Thank you for the valuable suggestion. The abstract has been improved now.

2. Comment: The introductory section needs to be significantly strengthened. It should begin with a focused discussion on phosphorus (P) chemistry and its dynamics in soil systems. Following this, the authors should clearly articulate how agronomic management practices—particularly varying tillage methods, amount of residue retention, and nitrogen (N) fertilization rates—affect soil P dynamics and availability, ultimately influencing crop growth and productivity. Establishing this context is essential to justify the research objectives and the significance of the study.

Author response: Thank you for this suggestion. We revised the manuscript and corrected the introduction section according to reviewer's valuable comments.

3. Comment: The most critical findings should be highlighted in result section. Special attention should be given to explaining the individual and interactive effects of tillage practices, crop residue levels, and N fertilization on phosphorus behavior and crop performance. If statistical interactions are observed, these must be explained clearly and meaningfully. The authors should interpret why and how such interactions occurred, grounded in sound agronomic or biochemical reasoning.

Author response: We appreciate the reviewer's feedback. The result section has been rewritten following the valuable comments of the reviewers.

4. Comment: The conclusion should be succinctly focused. It must restate the main findings of the study and their practical implications. Avoid reiterating background information or including new data. Instead, the conclusion should draw attention to the most relevant and impactful results and how they advance current understanding or practices.

Author response: Thank you for the valuable suggestion. As suggested by the reviewer, we have tried to improve the conclusion.

5. Comment: All data presented in tables must be systematically organized according to the experimental design, particularly reflecting the split-plot arrangement. Clear labeling, consistent formatting, and appropriate statistical indicators (e.g., LSD, SE, p-values) are essential to improve readability and interpretation.

Author response: Thank you for pointing these out. We made the necessary corrections in Tables following the comments of reviewers.
