# Supplementary material for: Long-term conservation agriculture with optimum nitrogen fertilization improves soil phosphorus availability
Source: PLoS One. 2025 Sep 24;20(9):e0333013. doi: 10.1371/journal.pone.0333013 (PMC12459793; doi:10.1371/journal.pone.0333013)
Supplement: Attachment — Submitted filename: Comments to authors a.docx [file pone.0333013.s002.docx]

**Comments to authors:**

Effect of long-term conservation agriculture with nitrogen rates on soil available and labile phosphorus pools

Manuscript Number: PONE-D-25-17789

**Detail suggestions are given below:**

1. Title: What type of “effect” should be specified? This is journal article not thesis.
2. Key words: Title words should not be kewords.
3. Abstract: Hypothesis is not clear and results should be measurable (% increase or decrease). Higher N rates did not reduce all P fractions.
4. Title words should not be key words.
5. Introduction: There are too many irrelevant topics included here. There are huge references on. But your references are too old, use latest references (Some link attached here ; <https://doi.org/10.1111/sum.13153>; <https://doi.org/10.1007/s42729-024-02009-z>; <https://doi.org/10.1007/s10705-024-10348-7>; <https://doi.org/10.1016/j.still.2022.105474>; <http://dx.doi.org/10.1007/s10705-023-10261-5>; <https://sssb1958.com/wp-content/uploads/2024/12/U-Kumar.pdf>). Lack of novelty and research scope. Introduction should be rearranged by the sequence of - important of the research, what is the known about the topic, unknown about the topic, why this research is important and finally aims of this research.

Line 112: AEZ 9 is Old Brahmaputra Floodplain not Old Himalayan Piedmont Plain.

6. Materials and methods: Line 144: Medium Highland Sonatala soil series is poorly drained soil not moderately drained.

Line 151-152: What are the crops diversifications? Provide the 12 years cropping pattern.

Line 162: RD? Elaborate first.

Line 168-171: Fertilizers doses for each crop are not clear. Provide in a table and give it to supplementary data.

Line 180-182: 0-15 soil depth? Sample collection procedure should be clear specially core sampler.

Line 186: descried by 29.?

7. Need to rewrite the results section. Results are not described appropriately. Several results are confusingly and repeatedly presented. If interaction is significant, present only interaction effect results in the table or figures. No need to describe main factor results. Result should be measurable e.g., how much increase or decrease must be specified. In general, several paragraphs are very repetitive of the information provided in Tables. It is recommended to highlight more important results, rather than repeat results presented in tables. Different P fractions have interlink with other soil properties like bulk density, texture, soil organic carbon, N, and extractable K, S, Zn, B, Ca, Mg, Fe, Mn, Cu etc. So, need to describe what is the relation among them? Crops yield is also need to describe to understand the optimum production with P fractions. The manuscript results has lack of novelty and unable to give specific message to the scientific communities.
8. Discussion is not another result. The discussion should link all of your findings. Each paragraph should be well structured and interconnected (“telling a story”). For example, in each paragraph of the discussion section, authors should begin with a sentence that introduces what the paragraph is about of their findings, link their findings with those in the literature, and finish with what are the main points ('take-home-message'). Authors also need to discuss about the limitations and lackings of their study and its justifications. Please, a deeper scientific interpretation of your findings in the discussion section is strongly suggested. The novelty and implication of your study should also be highlighted throughout this section. In addition, I suggest using more actualized.
9. The conclusions section should not be a summary of your study or an extension of the discussion or results. This section should illustrate the mechanistic links of findings obtained under applied treatments. The conclusions should answer the hypothesis of your study and should focus on the implication of your findings. Please, avoid using abbreviations and acronyms in this section. Remember that the conclusions must be self-explanatory. This section should highlight the novelty and implication of your study also.
10. Avoid unnecessary tables and figures and they must be self-explanatory.
11. English improvement is needed in whole MS

The purpose, novelty and originality of your study seem to be unclear and incomplete throughout the manuscript. The manuscript is unable to give new message to the scientific community. It is not acceptable in the present form. It requires substantial improvement in your manuscript.
